# Molecular hydrogen promotes retinal vascular regeneration and attenuates neovascularization and neuroglial dysfunction in oxygen-induced retinopathy mice

**DOI:** 10.1186/s40659-024-00515-z

**Published:** 2024-06-24

**Authors:** Yatu Guo, Jiahui Qin, Ruiqiang Sun, Peng Hao, Zhixin Jiang, Yuchuan Wang, Zhiqi Gao, Huan Zhang, Keliang Xie, Wei Zhang

**Affiliations:** 1 Tianjin Key Lab of Ophthalmology and Visual Science, Tianjin Eye Hospital, Tianjin, China; 2 Tianjin Eye Institute, Tianjin Eye Hospital, Tianjin, China; 3 Fenyang College of Shanxi Medical University, Shanxi, China; 4 Nankai University Affiliated Eye Hospital, Tianjin, China; 5 Department of Anesthesiology, General Hospital of Tianjin Medical University, Tianjin Research Institute of Anesthesiology, Tianjin, China; 6 Department of Critical Care Medicine, General Hospital of Tianjin Medical University, Tianjin, China

**Keywords:** OIR, Angiogenesis, neovascularization, Revascularization, H_2_, *Nrf2*, *Dll4*, *Notch*, *HIF-1α*, Retinal glia cell

## Abstract

**Background:**

Retinopathy of Prematurity (ROP) is a proliferative retinal vascular disease occurring in the retina of premature infants and is the main cause of childhood blindness. Nowadays anti-VEGF and retinal photocoagulation are mainstream treatments for ROP, but they develop a variety of complications. Hydrogen (H_2_) is widely considered as a useful neuroprotective and antioxidative therapeutic method for hypoxic-ischemic disease without toxic effects. However, whether H_2_ provides physiological angiogenesis promotion, neovascularization suppression and glial protection in the progression of ROP is largely unknown.This study aims to investigate the effects of H_2_ on retinal angiogenesis, neovascularization and neuroglial dysfunction in the retinas of oxygen-induced retinopathy (OIR) mice.

**Methods:**

In this study, mice that were seven days old and either wild-type (WT) or Nrf2-deficient (*Nrf2−/−*) were exposed to 75% oxygen for 5 days and then returned to normal air conditions. Different stages of hydrogen gas (H_2_) inhalation were administered. Vascular obliteration, neovascularization, and blood vessel leakage were analyzed and compared. To count the number of neovascularization endothelial nuclei, routine HE staining of retinal sections was conducted. Immunohistochemistry was performed using DyLight 594 labeled GSL I-isolectin B4 (IB4), as well as primary antibodies against proliferating cell nuclear antigen (PCNA), glial fibrillary acidic protein (GFAP), and Iba-1. Western blots were used to measure the expression of NF-E2-related factor 2 (*Nrf2*), vascular endothelial growth factor (*VEGF*), *Notch1*, *Dll4*, and *HIF-1α.* Additionally, the expression of target genes such as *NQO1, HO-1, Notch1, Hey1, Hey2*, and *Dll4* was measured. Human umbilical vein endothelial cells (HUVECs) treated with H_2_ under hypoxia were used as an in vitro model. RT-PCR was used to evaluate the mRNA expression of *Nrf2, Notch/Dll4*, and the target genes. The expression of reactive oxygen species (ROS) was observed using immunofluorescence staining.

**Results:**

Our results indicate that 3–4% H_2_ does not disturb retinal physiological angiogenesis, but ameliorates vaso-obliteration and neovascularization in OIR mice. Moreover, H_2_ prevents the decreased density and reverses the morphologic and functional changes in retinal astrocytes caused by oxygen-induced injury. In addition, H_2_ inhalation reduces microglial activation, especially in the area of neovascularization in OIR mice. H_2_ plays a protective role in vascular regeneration by promoting *Nrf2* activation and suppressing the *Dll4*-induced *Notch* signaling pathway in vivo. Also, H_2_ promotes the proliferation of HUVECs under hypoxia by negatively regulating the *Dll4/Notch* pathway and reducing ROS levels through *Nrf2* pathway aligning with our findings in vivo.Moreover, the retinal oxygen-sensing mechanisms (*HIF-1α/VEGF)* are also involved in hydrogen-mediated retinal revascularization and neovascularization suppression.

**Conclusions:**

Collectively, our results indicate that H_2_ could be a promising therapeutic agent for POR treatment and that its beneficial effect in human ROP might involve the activation of the *Nrf2-Notch* axis as well as *HIF-1α/VEGF* pathways.

**Graphical Abstract:**

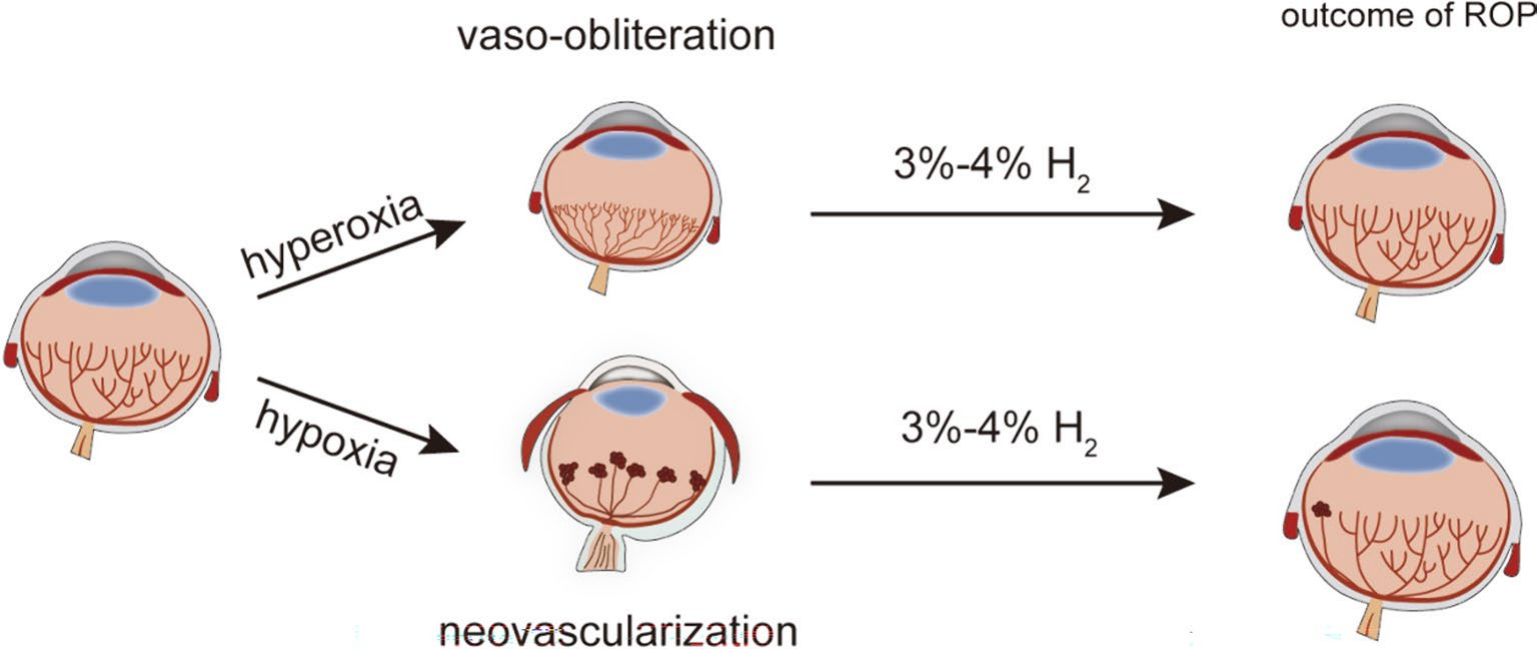

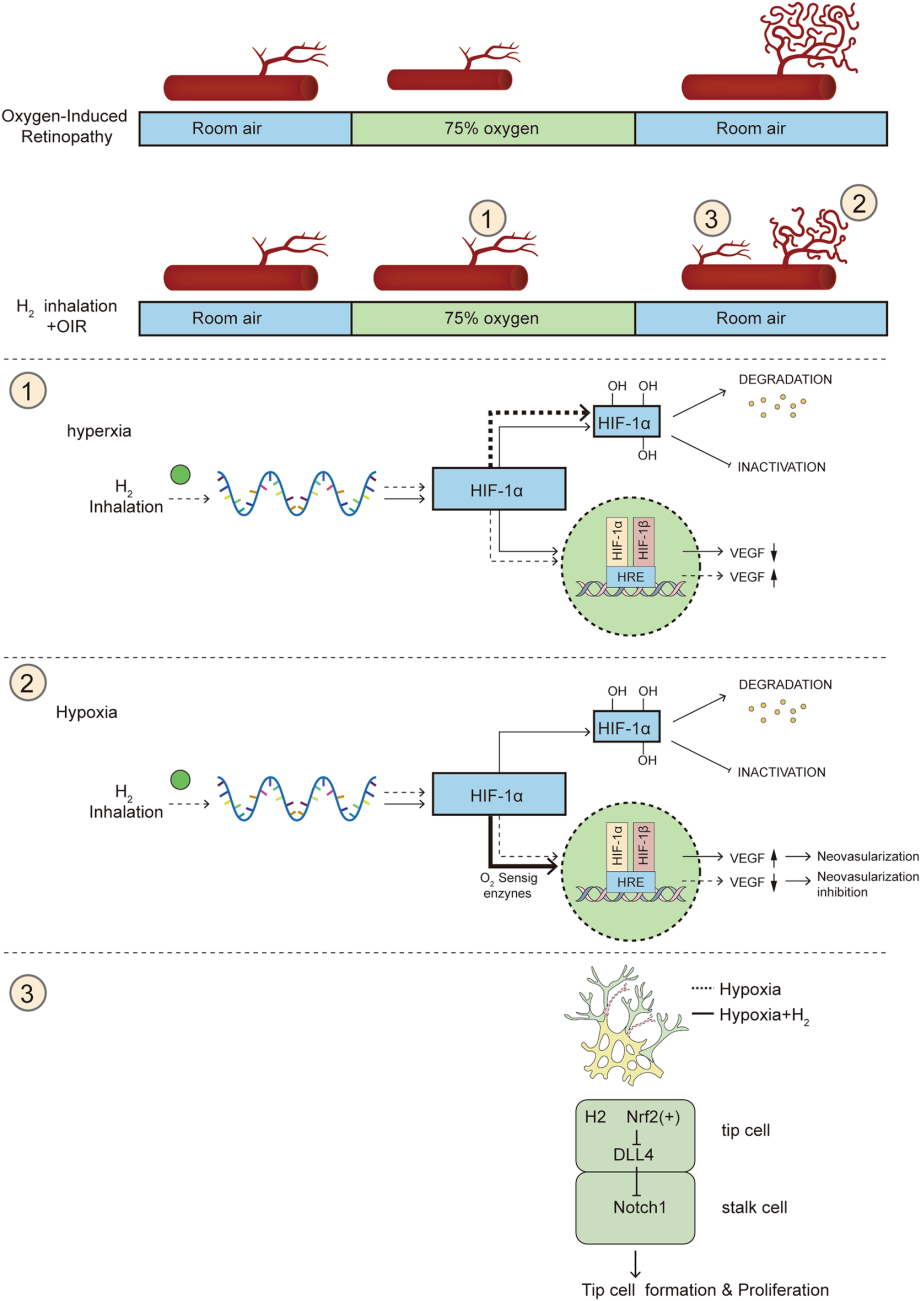

**Supplementary Information:**

The online version contains supplementary material available at 10.1186/s40659-024-00515-z.

## Introduction

Retinopathy of prematurity (ROP) is a major ocular disorder in premature infants and the leading cause of infantile blindness [1]. The disease has two postnatal phases. The inhibition of growth factor expression due to hyperoxia and disruption of the maternal–fetal interaction results in an arrest of retinal vascularization (phase I). Subsequently, in addition to increasingly active metabolism with poor vascularization, the retina becomes hypoxic after returning to a relatively hypoxic environment, stimulating vast proliferation induced by multiple factors (phase II), which leads to recurrent preretinal hemorrhage, fibrous scar formation, traction, and retinal detachment [2]. The latter phase is the vital factor contributing to ROP [3]. Oxidative stress and inflammation also play a role in the development of ROP, as an imbalance between the production and elimination of reactive oxygen species and inflammatory cytokines occurs [4]. To better understand the molecular mechanisms of ROP, researchers use a mouse model called oxygen-induced retinopathy (OIR), which mimics the clinical features of ROP [5].

As the most effective treatment for ROP, laser therapy has primarily replaced cryotherapy in current practice [6]. Unfortunately, these treatments have the disadvantages of permanent loss of peripheral vision and high incidence of myopia [7]. It is widely known that vascular endothelial growth factor (VEGF) is a pivotal regulator of physiological and pathological angiogenesis. Numerous studies support that angiogenic factors such as VEGF cause phases of ROP [8]. Therefore, as an alternative, anti-vascular endothelial growth factor agent (anti-VEGF) therapy is also a promising treatment for ROP [9]. However, using anti-VEGF agents, especially in premature infants, is associated with a high recurrence rate and potential systemic side effects. Immature retinas are more susceptible to VEGF, which causes blood-eye barrier (BRB) breakdown [10]. More seriously, it may enter the systemic circulation and inhibit the VEGF receptor in neonatal vital organs [11, 12]. The therapeutic limitations show an urgent need for new therapeutic strategies to treat or prevent ROP with higher efficacy to reduce recurrence. Treatments to enhance physiological retinal vascular development without side effects have been hot topics in recent research.

In the past decade, molecular hydrogen (H_2_) has been recognized as a novel therapeutic medical gas. In 2007, Ohsawa et al. identified H_2_-induced protective effects in a rat model of cerebral infarction and highlighted its ability to scavenge gaseous radicals [13]. H_2_ has unique properties, including its rapid diffusion into tissues, ability to penetrate through blood‒brain and blood‒retina barriers, and selectively scavenges highly toxic ROS, particularly hydroxyl radicals and peroxynitrite [14]. At the cellular level, H_2_ can enter mitochondria and even translocate to the nucleus under certain conditions.In addition to its ROS scavenging abilities, H_2_ has been found to possess anti-apoptotic, cytoprotective, and anti-inflammatory properties. Notably, H_2_ can easily penetrate target tissues and cells through gaseous diffusion without affecting physiological parameters like pH or oxygen saturation [15]. These unique characteristics have led to the exploration of H_2_ as a potential therapeutic agent for ocular diseases, particularly those affecting the retina. Studies using phototoxic models have shown that H_2_ can effectively scavenge free radicals and alleviate oxidative stress, thereby preventing retinal damage in conditions such as age-related macular degeneration, retinitis pigmentosa, and diabetic retinopathy. H_2_ has also demonstrated positive effects in managing traumatic optic neuropathy and slowing the formation of cataracts by preserving antioxidant capacity. Additionally, H_2_ shows promise as a potential treatment for corneal alkali injury by reducing NF-kappa B phosphorylation and VEGF protein levels. Given its potent antioxidant, anti-inflammatory activity, and favorable safety profile, H_2_ may serve as a valuable therapeutic approach for retinopathy of prematurity (ROP) and other ocular pathologies [16].

NF-E2-related factor 2 (*Nrf2*) is a major stress response transcription factor known for its cell-intrinsic cytoprotective function. *Nrf2* is best known as a master regulator governing redox homeostasis in mammalian cells [17, 18]. Previous studies reported that the activation of *Nrf2* protected the retina against oxidative stress and inflammatory responses [19]. Additionally, *Nrf2* directly affects retinal angiogenesis in both normal and ischemic retinas [20]. *Nrf2* signaling has thus become a promising target for potential therapeutic agents for ROP. Molecular hydrogen (H_2_) has antioxidant and anti-inflammatory properties that are closely linked to the activation of *Nrf2* signaling [21, 22]. The Notch pathway, present in mammals, has diverse effects on various tissues in the body. It regulates many embryonic and postnatal development events, including proliferation, apoptosis, border formation, and cell fate decisions. Specifically, vascular sprouting suppression results from increased *Dll4/Notch* signaling. The activation of *Nrf2* plays a crucial role in modulating *Dll4/Notch* signaling, which is necessary for promoting the growth of new blood vessels [20]. In addition, several studies have suggested that hypoxia-inducible factor-1α (*Hif-1α*) is stabilized and accumulates, which is attributed to elevated ROS during hypoxia. Subsequently, it translocates to the nucleus, binding constitutively expressed β-subunit (*Hif-1β*), forming HIF-1 [21]. *HIF-1* binds hypoxia response elements on numerous genes encoding proteins necessary for responding to hypoxic stress, such as VEGF [23].

Given the above, we aimed to investigate the hypothesis that H_2_ inhalation alleviates vaso-obliteration and neovascularization and promotes revascularization of the retina through the *Nrf2-notch* axis modulation and *HIF-1α/VEGF* pathways inhibition in OIR mice.

## Materials and methods

### Reagents

Bovine serum albumin (BSA), phosphate-buffered saline (PBS) solution and DyLight 594 LAabeled GSL I-isolectin B4 were purchased from Shanghai Maokang Biotechnology Co., Ltd. (MIKBio, Shanghai, China). Antibodies against GFAP, VEGFA, and Noth1 were purchased from Cell Signaling Technology (Danvers, MA, USA). Proliferating cell nuclear antigen (PCNA) and β-actin were purchased from Cell Signaling (Beverly, CA, USA). Antibodies against Nrf2, HIF-1α and Iba1 were purchased from Wako Pure Chemical Industries (Abcam, UK). Antibodies against Dll4 were purchased from R&D Systems (Minneapolis, MN, USA). The optimum cutting temperature (O.C.T.) compound was purchased from Sakura Finetek (Torrance, CA, USA).

### Mice and OIR mouse model

Specific pathogen-free male mice (C57BL/6J, wild-type(WT), and *Nrf2−/−*) were used in this study. All *Nrf2−/−* mice had the same genetic background as C57BL/6J mice.C57Bl/6J mice were purchased from Beijing Vital River Laboratories Co., Ltd. [SCXK (JING) 2016-0006, Beijing, China]. *Nrf2−/−* mice (C57BL/6-Nf^e2l2em1Smoc^)were purchased from Shanghai Model Organisms. The OIR mouse model was generated as described in a previous report [4]. Briefly, the newborn mice at P7 and their nursing mothers were exposed to 75% oxygen for 5 days and then were returned to room air (21% oxygen) for 5 days at P12. All animal experiments were performed according to the Association for Research in Vision and Ophthalmology (ARVO) statement for the use of Animals in Ophthalmic and Vision Research and protocols approved by the Institutional Animal Care and Use Committee at Nankai University (Approval No.NKYY-DWLL-2021-070). All animals were bred and housed in our pathogen-free animal facility and were fed a standard normal diet ad libitum with free access to water.

**Fig. 1:**
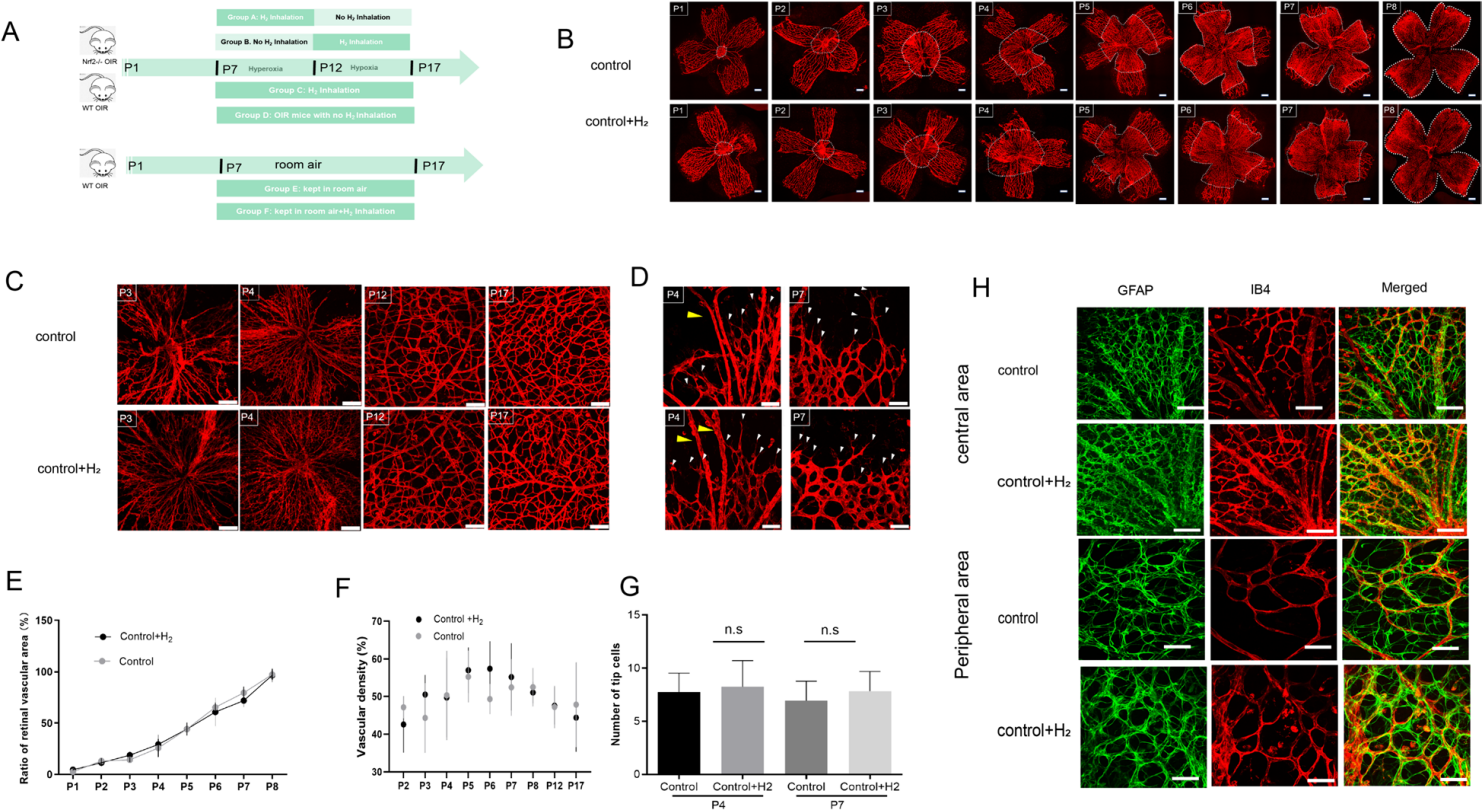
H2 inhalation does not interrupt retinal physiologic vascularization during postnatal development. **A** .The schematic diagram of animal experiment. **B**-**C**. H2 inhalation does not interrupt retinal physiologic vascularization. **D**-**H**. The retinal distributions of astrocytes and the number of tip cells at P4 and P7. **E**-**G**. The ratio of retinal vascular area(**E**), vascular density (**F**) and number of tip cells were quantified and analyzed. Two-way ANOVA: control group vs. control group; Control+H2 F(1,16) =0.305 P= 0.5881;n=8 each group Error bars were the mean ± SEM. *p < 0.05

Both *Nrf2−/−* OIR mice and WT OIR mice were divided into four groups: Group A:H_2_ inhalation from P7 to P12; Group B:H_2_ inhalation from P12 to P17; Group C:H_2_ inhalation from P7 to P17; Group D: OIR mice with no intervention.In addition, Group E was considered as the control group, in which the WT mice were kept in room air (21% oxygen) during the whole process. Group F was designed to evaluate the effect of H_2_ on normal retinal angiogenesis.Each group consists of a minimum of 10 to 12 mice (see Supplementary Table 1). WT mice were kept in room air (21% oxygen) with H_2_ inhalation (3–4%) fromP7 to P17 (3–4% H_2_ was inhaled for 6 h per day, shown in Fig. 1A) The concentration of O_2_ and H_2_ were monitored oxygen chamber (Tow-INT TECH Model ProOx-850, Shanghai, CN).

### Whole-mounted retinal immunofluorescence staining

Mice were euthanized at P12 or P17, and eyes were enucleated and fixed overnight in 4% paraformaldehyde (PFA). After being dissected under an operating microscope, intact retinas were blocked and permeabilized in PBS containing 5% BSA and 0.3% Triton-X-100 overnight at 4 °C. Then, retinas were incubated with primary antibodies against IB4 (1:100), GFAP (1:200), or Iba1 (1:200) overnight at 4 °C. After washing with PBS, a mixture of fluorescein isothiocyanate (FITC)- conjugated secondary antibodies was added and incubated for 2 h at room temperature. After washing with PBS, the retinas were flat mounted and analyzed by confocal microscopy (Leica TCS SP8 SMD). Retinal vaso-obliteration and pathologic angiogenesis were quantified as described earlier [18]. In short, the formation of neovascularization clusters was quantified by comparing the number of pixels in the affected area with the number of pixels in the retina. Microglia, astrocytes and tip cells were all counted manually using Photoshop. Measurement were performed blind to the identity of the sample.

### Retinal frozen-section immunofluorescence

To generate frozen retinal sections, the eyes of the mice were enucleated and fixed overnight in 4% PFA. Next, eyes were dehydrated in 30% sucrose and embedded in O.C.T. compound. Then, they were fast frozen and cut into 8-μm-thick sections. The sections were washed with PBS and then permeabilized with 1% Triton-X-100 for 15 min at room temperature (RT) before blocking with 1% BSA for 2 h. The sections were incubated with DyLight 594 LAabeled GSL I-isolectin B4 (IB4, 1:100) and primary antibodies against PCNA (1:200), *Nrf2* (1:100) and *HIF-1α* (1:100) overnight at 4 °C. After washing in PBS, sections were incubated for 2 h at RT with a mixture of fluorescein isothiocyanate of FITC- conjugated secondary antibodies and counterstained with DAPI for 15 min. The frozen retinal sections were then analyzed using confocal microscopy (Leica TCS SP8 SMD).

### HE staining

Routine HE staining of retinal sections was performed to count the number of neovascularization endothelial nuclei breaking through the inner boundary membrane of the retina to quantitatively reflect the hyperplasia of retinal vessels [19]. The eyeball was removed, fixed in 4% paraformaldehyde for 24 h and embedded in paraffin. A 6-micron continuous section was dissected parallel to the optic nerve and stained with hematoxylin and eosin. The retinal vasculature was assessed by light microscopy (Olympus, Germany, × 400). The preretinal neovascular nuclei in the eye were counted.

### Cell culture

In a 37 ℃, 5% CO_2_ incubator, human umbilical vein endothelial cells(HUVECs) were grown. 10% fetal bovine serum(Gibco BRL, Gaethersburg, USA), 1% penicillin–streptomycin solution, and culture medium (Thermo Fisher RPMI 1640, Waltham, USA) make up the total culture media. Building cell models from 2nd to 6th generation cells. To induce hypoxic injury, HUVECs were exposed to low oxygen-containing gas (94% N_2_, 5% CO_2_, and 1% O_2_) for 24 h, as previously reported [24]. The cells were split into five groups: normal, control (NC), hypoxic (hypoxic) (94% N_2_, 5% CO_2_, 1% O_2_, 24 h), hypoxic + hydrogen (95% N_2_, 4% CO_2_, 1% O_2_, 24 h + H_2_, 2 h), and hypoxic + hydrogen with *Nrf2* inhibition (hypoxia + H_2_ + ML385) (95% N_2_, 4% CO_2_, 1% O_2_, 24 h + H_2_, 2 h + ML385 2 μm, 24 h) (Selleck, Shanghai, China).

### Determination of cell viability

Utilize the MTT assay to gauge cell viability (Solarbio, Beijing, China). Plant HUVEC in a 96-well plate, then starve them for the night after 12 h of incubation. Under hypoxic, hypoxic plus hydrogen, and hypoxic plus hydrogen plus *Nrf2* inhibition conditions, respectively, incubate. Then, DMSO was added and left to stand for 15 min after adding the MTT response for 4 h. Use an enzyme-linked immunosorbent assay(Thermo 3001 VARIOSKAN FLASH, California, USA) to determine the optical density (OD) value at 490 nm.

### Transwell

Making a cell suspension: Cells are digested by pancreatin, which also prepares the cell suspension, inserts the top chamber into a well plate, and arranges the cells there. In the lower chamber, add a culture media containing 20% serum. Cultivate in a cell incubator for 16 h. For 20 min, fix cells with 4% paraformaldehyde. To count the number of cells migrating, stain the upper compartment with crystal violet, wipe the cells clean using a cotton swab, and take pictures.

### ROS

After the modelling is finished, the cells are laid, and the operation is not started until the density reaches 70%. After removing the cell culture medium and the inducing medication, dilute the DCFH-DA working solution with serum-free culture medium at a ratio of 1:1000 (made in Nanjing), and then incubate the cells for 20 min with the diluted DCFH-DA solution. To eliminate DCFH-DA that has not penetrated the cells, wash the cells once or twice with serum-free culture media. Subsequently, confocal imaging is applied to photography.

### Protein extraction and western blot analysis

Retinas were removed from mice of each group at P12 or P17. Protein was extracted from the retinas with RIPA containing protease inhibitors. Protein concentrations were determined by the Bradford method using BSA as a standard. Samples of supernatants containing 20 μg protein were heated to 95 °C for 10 min and separated by sodium dodecyl sulfate-PAGE and transferred to polyvinylidine difluoride filter (PVDF) membranes (Merck Millipore, Suzhou Heyi Biotech Co. Ltd., Suzhou, China). After blocking with 5% defatted milk in PBS-Tween-20 for 1 h at room temperature, the PVDF membranes were incubated with diluted Nrf2 (1:1000), Notch1 (1:1000), Dll4 (1:1000), HIF-1α (1:1000), VEGFA (1:1000), GAPDH (1:5000), β-Tubulin (1:5000), GAPDH (1:5000) and β-actin (1:5000) antibodies in blocking solution overnight at 4 °C. On the following day, after washing for three times, the PVDF membranes were then incubated with the secondary antibody, HRP (1:10,000) for 2 h and visualized using an enhanced chemiluminescence system (Millipore).

### Quantitative real-time PCR

Utilize the EZB kit (B0004D, EZ Bioscience) to extract total RNA. Using the abm qPCR RT kit (G592, abm, BC, Canada), convert 1 µg of total RNA into cDNA. Use the Roche LightCycle 96 qPCR system and SYBR Green FastStart 2X Master Mix (abm, Richmond, BC, Canada) to perform qPCR. Expression of target genes was measured in triplicate and was normalized to β-actin expression as an internal control. The 2^−∆∆Cq^ method was used to calculate target gene expression. The Table 1 displays the primer sequences that were employed.

**Table 1:** Primer sequences

Gene	Species	PubMed ID	→ Sequence(5′ 3′)
β-Actin	human	NM_001101.5	F: CCTTCCTTCCTGGGCATGG
			R: TCTGCATCCTGTCGGCAATG
Nrf2	human	NM_001313904.1	F: GTGCTGTCAAGGGACATGGA
			R: AGTGACTGAAACGTAGCCGAA
DLL4	human	NM_019074.4	F: CCATGCAAGAATGGGGCAAC
			R: GGCCATCCTCCTGGTCCTTAC
Notch1	human	NM_017617.5	F: TGGACCAGATTGGGGAGTTC
			R: GCACACTCGTCTGTGTTGAC
NQ-1	human	NM_001025433.2	F: TATCCTGCCGAGTCTGTTCTG
			R: AACTGGAATATCACAAGGTCTGC
HO-1	human	NM_002133.3	F: AAGACTGCGTTCCTGCTCAA
			R: GGGGGCAGAATCTTGCACT
β-Actin	Mouse	NM_007393.5	F: GTGACGTTGACATCCGTAAAGA
			R: GCCGGACTCATCGTACTCC
NQO1	Mouse	NM_008706.5	F: ATGGGAGGTGGTCGAATCTGA
			R: GCCTTCCTTATACGCCAGAGATG
HO-1	Mouse	NM_010442.2	F: AAGCCGAGAATGCTGAGTTCA
			R: GCCGTGTAGATATGGTACAAGGA
Notch1	Mouse	NM_008714.3	F: GATGGCCTCAATGGGTACAAG
			R: TCGTTGTTGTTGATGTCACAGT
Hey1	Mouse	NM_010423.2	F: GCGCGGACGAGAATGGAAA
			R: TCAGGTGATCCACAGTCATCTG
Hes1	Mouse	NM_001416728.1	F: CCAGCCAGTGTCAACACGA
			R: AATGCCGGGAGCTATCTTTCT
Dll4	Mouse	NM_019454.4	F: TTCCAGGCAACCTTCTCCGA
			R: ACTGCCGCTATTCTTGTCCC

### Statistical analysis

Data are presented as mean ± SEM for normally distributed continuous variables, median and interquartile range for non-normally distributed continuous variables, and number (%) for categorical variables. The normality and homogeneity of variance were assessed using the Kolmogorov–Smirnov test. Student’s t-test was applied for normally distributed data, and the Mann–Whitney U test was used for non-normally distributed data in two-group comparisons. For analyses involving multiple groups, one-way ANOVA with Bonferroni’s correction was utilized for continuous variables, while the Chi-square test was employed for categorical variables. Statistical significance was set at P < 0.05. All statistical analyses were performed using GraphPad Prism software (GraphPad Prism 5, GraphPad Software, Inc., San Diego, CA, USA).

## Results

### H_2_ inhalation does no disrupt retinal physiologic vascularization during postnatal development

To investigate the effect of H_2_ on the formation and maturation of the developmental retinal vascular network, we investigated developmental angiogenesis in the retina in H_2_-treated WT mice and untreated littermate controls from P1 to P17.

As shown in Fig. 1B and C, after birth, the retinal vascular system starts to develop as a sprout from the optic disc and initially forms a primitive vascular plexus that is rapidly remodeled into large and small vessels.By around P8, retinal vessels extend radially over the superficial layer of the retina to form a two-dimensional vascular structure and subsequently sprout into the retina, establishing a secondary, deeper plexus. To assess retinal vascularization, we measured the ratio of the retinal vascular area (area of retinal blood vessels within the white dotted line) to the area of the entire retina [22]. The mice inhaling H_2_ (concentration: 3–4%) displayed normal primary vascular plexus and vascular density, similar to littermate controls, indicating that H_2_ inhalation does not interfere with the normal extension of retinal blood vessels and the density of the vascular network (Fig. 1F, E).

Endothelial tip cells are leading cells at the tips of the area undergoing vascular sprouting during angiogenesis. Additionally, retinal astrocytes are found in species that possess a retinal vasculature, providing the foundation for retinal angiogenesis and guiding the migration of endothelial tip cells. We examined the retinal distribution of astrocytes and the number of tip cells at P4 and P7, as shown in Fig. 1D and H. Mice that underwent H_2_ inhalation and controls showed no differences in tip cells at P4 and P7 (Fig. 1G). Moreover, normal distribution, morphology, and densities of glial fibrillary acidic protein (GFAP) expression in astrocytes were observed in both the central and peripheral retinas at P7 and P17 (Fig. 1H). Notably, the complete formation of a three-layered vascular system was detected in both groups at P17 through 3D image reconstruction by confocal laser scanning microscopy (Figure S1). Altogether, the results indicate that H_2_ does not interfere with retinal angiogenesis during postnatal development.

### H_2_ attenuates retinal vaso-obliteration and neovascularization in OIR mice

**Fig. 2:**
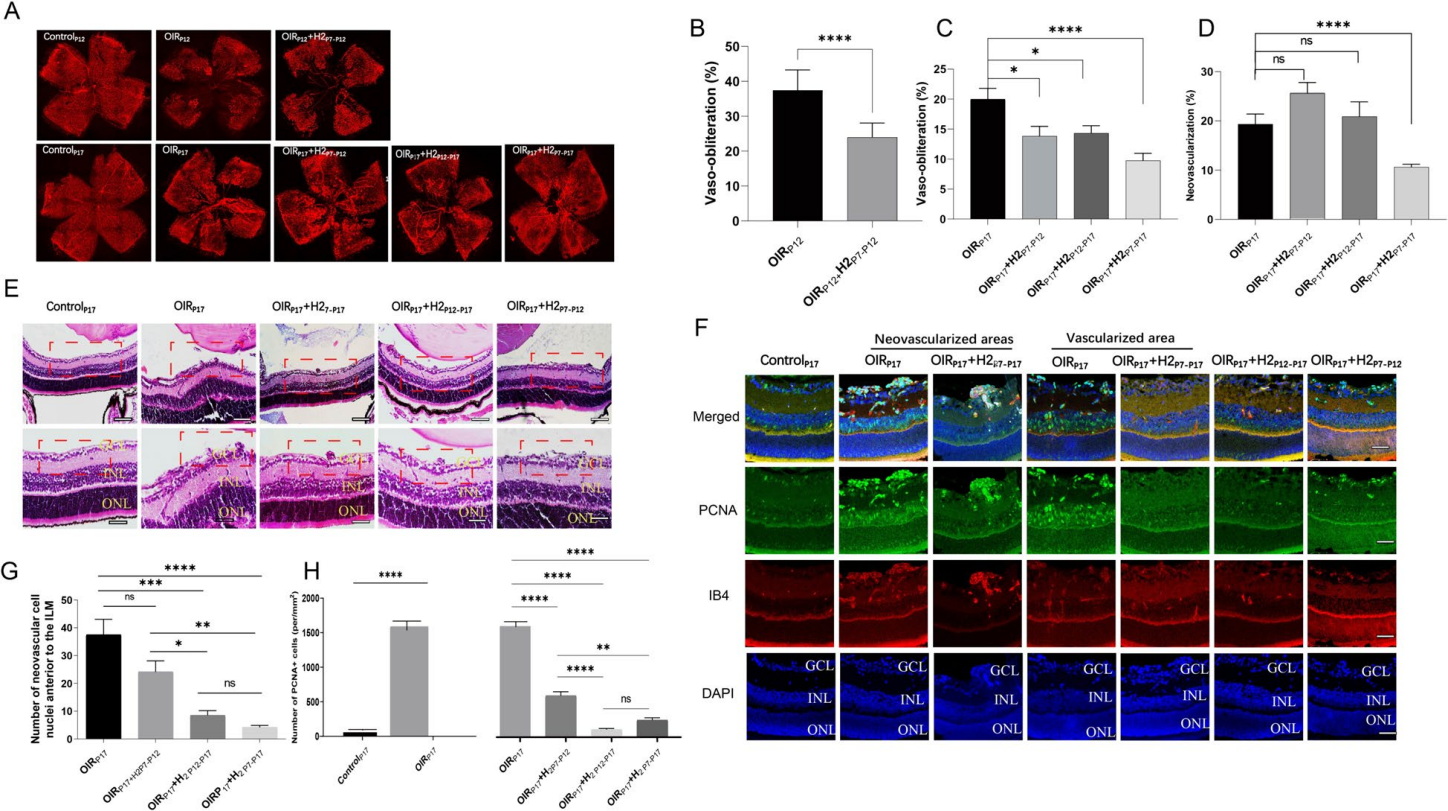
H2 attenuates retinal vaso-obliteration and neovascularization in OIR mice. **A.** Whole-mount retinas from the Control, OIR and OIR+H2 groups of wild type mice were harvested on P12 or 17 and examined by immunostaining with isolectin B4 (bar=500μm). H2 treatment reduces retinal vaso-obliteration at P12 and pathologic neovascularization at P17 in OIR. Areas of vaso-obliteration (**B**, **C**) and neovascularization (**D**) were quantified and analyzed. Error bars were the mean ± SEM. *P < 0.05 n=10-15/group. **E**-**G**. H2 resulted in a decrease in endothelial cell nuclei numbers anterior to the inner limiting membrane in OIR mice. **F**. The presence of PCNA-positive cells was confined to the retinal vessel area after H2 inhalation

We utilized a mouse model of oxygen-induced retinopathy (OIR), where mice were exposed to 75% oxygen from P7 to P12 and returned to room air until P17 [4]. The hyperoxic phase (P7–P12) results in vaso-obliteration of the central retina. This phase is followed by a second phase (P12–P17) of vascular regeneration in this central avascular retina (Fig. 1A). To gain insights into the role of H_2_ role in regulating vascularization in the retina, we examined the changes in OIR mice retinas with H_2_ inhalation on P12 and P17. As shown in Fig. 2A, OIR mice displayed a 37.44% ± 5.56% higher avascular area compared to the control group during the hyperoxic period P7–P12). After five days of H_2_ inhalation from P7 to P12, the avascular area in OIR mouse retinas decreased to 23.96% ± 3.97% (Fig. 2B). Subsequently, we extended the H_2_ treatment for the entire period. At P17, when the OIR mice were in a hypoxic state, a neovascular tuft (NVT) formed due to retinal vessel occlusion, resulting in an avascular area of 19.98% ± 6.30% and an NVT area of 18.34% ± 6.56%. However, H_2_ dramatically reduced the avascular area to 9.77% ± 4.49% after the complete treatment, while simultaneously suppressing NVT area formation by 10.63% ± 2.23% (Fig. 2C, D). It is noteworthy that the use of H_2_ led to an reduction in vaso-obliteration during both hyperoxic and hypoxic phases, whether it was administered solely during those phases or throughout the entire treatment (Fig. 2A–C). However, inhalation of H_2_ only during the hypo or hyperoxic phase did not yield as favorable results as full treatment with H_2_ on neovascularization, as the latter significantly decreased neovascularization (Fig. 2D).

To evaluate the degree of retinal neovascularization accurately, we opted for the evaluation of neovascular cell nuclei located anterior to the internal limiting membrane (ILM) as an indicator, due to the imprecise nature of assessing retinal neovascularization using the retinal vessel ratio.At P17,retinas from the OIR_P17_ group contained multiple NVTs extending into the vitreous. In contrast, inhalation of H_2_ resulted in a decrease in endothelial cell nuclei numbers anterior to the inner limiting membrane, with inhibitory effect of 64.4%, 77.17% and 88.67% reduction in the OIR_P17_ + H_2P7–P12,_ OIR_P17_ + H_2P12–P17_ group and OIR_P17_ + H_2P7–P17_ group,respectively (Fig. 2E, G). These findings demonstrate that H_2_ inhalation suppressed pathological angiogenesis in the retina, preventing it from extending into the vitreous through the ILM.

In addition, proliferating cell nuclear antigen (PCNA), which is crucial for DNA replication and acts as a processivity factor for DNA polymerase δ in eukaryotic cells, is closely linked to the proliferation of vascular endothelial cells [25]. As depicted in Fig. 2F, the control group exhibited lower levels of PCNA expression, while PCNA-positive cells were mainly found in the retinal ganglion cell layer (GCL), inner nuclear layer (INL), and the vascular distribution region of the retina in the OIR_P17_ group, regardless of whether it was the neovascularization zone or the normal vessel area. Within the neovascular area of the OIR_P17_ group, the number of PCNA cells increased significantly, surpassing the control group by more than 20-fold. Following inhalation of H_2_, the presence of PCNA-positive cells was confined to the retinal vessel area. This led to reductions of 85.91%, 60.81%, and 92.94% in the OIR_P17+H2P7–P17_, OIR_P17+H2P7-P12_, and OIR_P17+H2P12–P17_ groups, respectively (Fig. 2F, H).These results indicated that in response to increased DNA damage and breaks caused by hypoxia and ischemia, the expression of PCNA is elevated to adapt to these changes.H_2_ demonstrated its efficacy in reducing abnormal blood vessel growth by decreasing the proliferation of vessels positive for PCNA during the hyperoxic or hypoxic phases, or throughout the entire treatment period.

### H_2_ preserves the astrocytic template and inhibits microgliosis in the retina of OIR mice

**Fig. 3:**
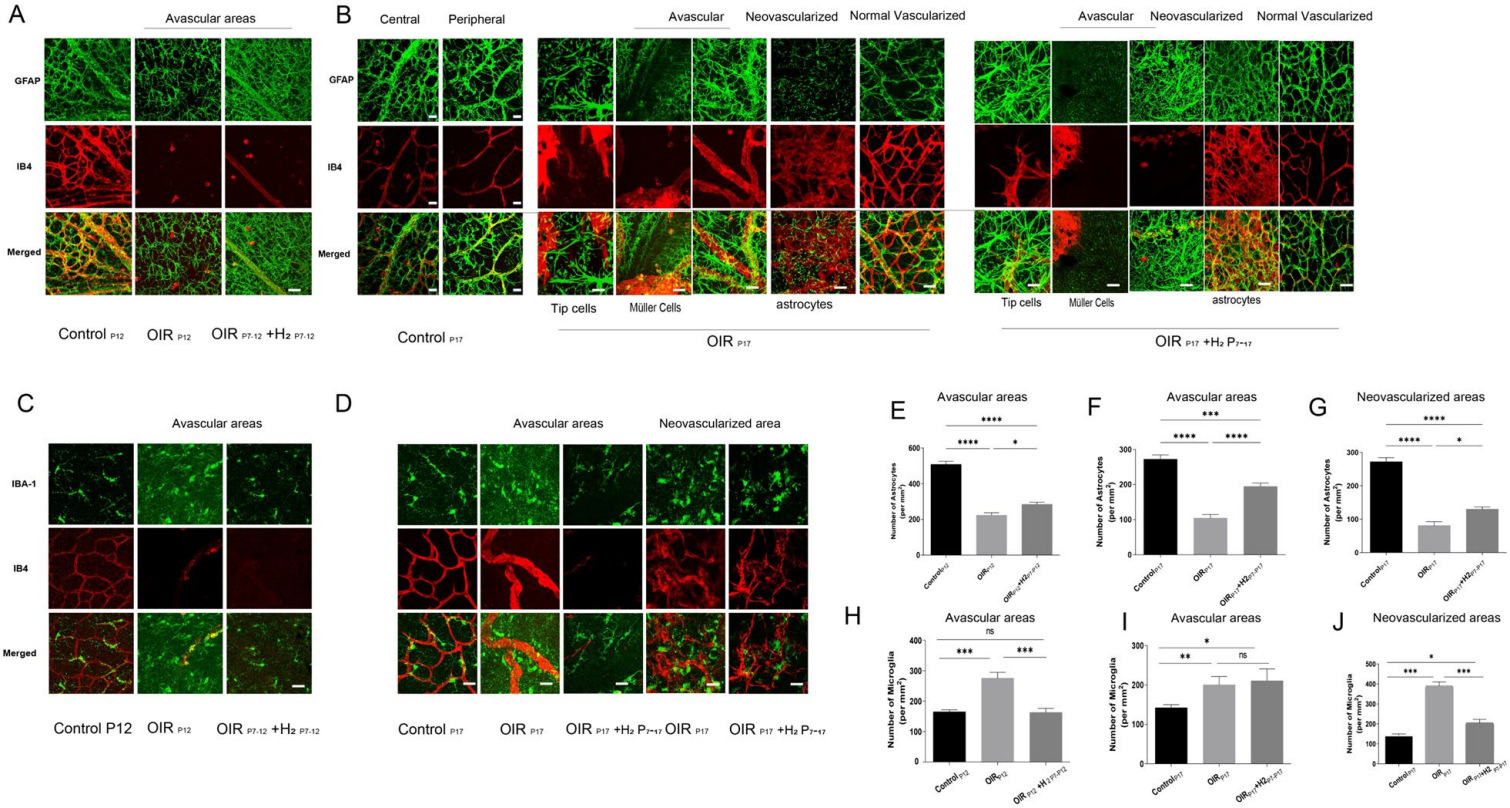
H2 preserves the astrocytic template and inhibits microgliosis in the retina of OIR mice. **A.E** H2 preserved the astrocytic template for the retinal vasculature in the avascular area during the hyperoxic period. **B.F.G** H2 treatment maintained the integrity and arrangement of astrocytes, effectively minimizing the disturbance of the astrocyte template in both areas of retinal avascularity and abnormal blood vessel growth in OIR retinasduring the hypoxic phase. **C.D H-J**. In OIR mice, microglia with ameboid and ramified forms were observed throughout the entire retina at P12 and P17; inhalation of H2 in OIR mice resulted in a reduction in the proportion of ameboid microglial cells compared to the OIR group at P12, with minimal changes observed at P17 in the avascular area. Error bars were the mean ± SEM. *P < 0.05

To understand the impact of H_2_ on glial cells in OIR mouse retinas [19], we examined the changes in astrocytes and microglia after H_2_ inhalation on P12 and P17. We investigated the distribution, cell density, and morphology of astrocytes labeled by GFAP immunostaining to assess the evolution of astrocyte changes. Normally, astrocytes are located between the vasculature and retinal neurons and are mostly found in the retinal nerve fiber layer (RNFL). Figure 3A illustrates the distribution and cell density of astrocytes in the avascular area of the retinas on P12. In the OIR mouse retinas, there was a 43.92% reduction in astrocytes compared to the control group, although the right morphology was maintained. However, H_2_ treatment improved the density of astrocytes to 56.02% of the control in the avascular area (Fig. 3A, E). These findings suggest that H2 preserved the astrocytic template for the retinal vasculature in the avascular area during the hyperoxic period.

During the hypoxic phase, astrocytes in the avascular areas of OIR_P17_ mouse retinas exhibited reduced ramification, hypertrophy, and disorganization compared to the control groups. The number of astrocytes was also significantly decreased, representing only 38.49% of the control group (Fig. 3B, F). In the neovascular area, the density of astrocytes in the OIR group dropped even further, reaching only 29.85% of the control group. The morphology and distribution of astrocytes were particularly disrupted in areas with neovascular tufts (Fig. 3B, G). However, in the avascular areas of OIR_P17_ + H_2P7–P17_ mouse retinas, the morphology of astrocytes was similar to that of OIR_P17_ mice, but the cell density increased to 71.62% of the control group after the entire treatment period. Furthermore, in contrast to the poorly distinguishable and irregularly arranged astrocytes mixed with neovascular tufts in OIR_P17_ mice, the astrocytes in the neovascular area of the OIR_P17_ + H_2 P7–P17_ group showed a reticular arrangement surrounding vessels, and the density increased to 47.83% of the normal control group (Fig. 3B, F, G). Additionally, in both the OIR_P17_ and OIR_P17_ + H_2 P7–P17_ groups, astrocytes were normally distributed in the area with normal vascular distribution (Fig. 3B). The findings demonstrated that H_2_ treatment maintained the integrity and arrangement of astrocytes, effectively minimizing the disturbance of the astrocyte template in both areas of retinal avascularity and abnormal blood vessel growth in OIR retinas.

We then examined the relationship between astrocytes and tip cells, which are leading cells in the sprouting of blood vessels. Immunostaining was performed to determine the colocalization of astrocytes and tip cells using GFAP and IB4. In the avascular and neovascularized regions of OIR_P17_ mouse retinas, there was either no colocalization or disordered colocalization between IB4 and GFAP, as shown in Fig. 3B. In contrast, a significant degree of colocalization was observed between anti-GFAP staining and IB-4 fluorescence in the avascular and neovascularized zones of OIR_P17_ + H_2 P7–P17_ mouse retinas. This suggests that astrocytes played a crucial role in guiding the tip cells in the OIR_P17_ + H_2 P7–P17_ group, as evidenced by the attachment of tip cells to astrocytes and the extension of their filopodia along the astrocytes (Fig. 3E–G). These findings indicate that H_2_ treatment maintained the quantity, morphology, and distribution of astrocytes, preserving the astrocyte template in OIR mouse retinas. Furthermore, our data suggest that H_2_ treatment improved the formation of tip cells, guided by the astrocyte network.

Furthermore, multiple studies have demonstrated the involvement of microglia in normal retinal vascular development [21]. Immunohistochemical analysis of the Iba-1 antigen on postnatal day 12 (P12) and P17 whole-mount retinas of normal mice revealed that microglia exhibited small cell bodies and long/thin cellular processes (Fig. 3C and D). In OIR mice, isolectin-immunopositive microglia with ameboid and ramified forms were observed throughout the entire retina at P12 and P17. The density of activated ameboid microglia in both the avascular and neovascular areas of OIR retinas was significantly higher than that in the control group, while the density of the nonactivated dendritic phenotype remained relatively unchanged. In contrast to OIR_P12_ mice, microglial cells in the peripheral neovascular area of OIR_P17_ mice tended to cluster near the neovascular tufts (NVTs) and exhibited enlarged cell bodies with shorter and fewer branched processes (Fig. 3D, H–J). Interestingly, inhalation of H_2_ in OIR mice resulted in a 59.1% reduction in the proportion of ameboid microglial cells compared to the OIR group at P12, with minimal changes observed at P17 in the avascular area, while the density of the dendritic phenotype remained unaffected (Fig. 3H–J). Moreover, treatment with H_2_ in OIR mice led to a reduction of approximately 52.33% in the proportion of ameboid microglial cells in the neovascular area, accompanied by a decrease in NVT size (Fig. 3J). These findings clearly show that H_2_ treatment inhibits microglial activation in the retinas of OIR mice, demonstrating the protective effect of H_2_ on neuroglial dysfunction in the OIR model.

### H_2_ promotes *Nrf2* activation in OIR mice

*Nrf2* has been found to regulate retinal angiogenesis [20]. To gain insights into the role of *Nrf2* and the relationship between H_2_ and *Nrf2* in the regulation of vascular regeneration in ischemic neuronal tissue, we first investigated the expression of *Nrf2* using quantitative PCR, immunoblotting, and immunofluorescence staining. *Nrf2* expression could be observed in the INL and GCL of the normal retina, where vessels and neurons are mainly located. We found that the expression of *Nrf2* in the OIR_P12_ group was the same as that in the control group in the avascular and normal blood vessel areas. However, in the OIR_P17_ group, *Nrf2* expression in the neovascular and normal vascularized areas was higher than in the control group.

**Fig. 4:**
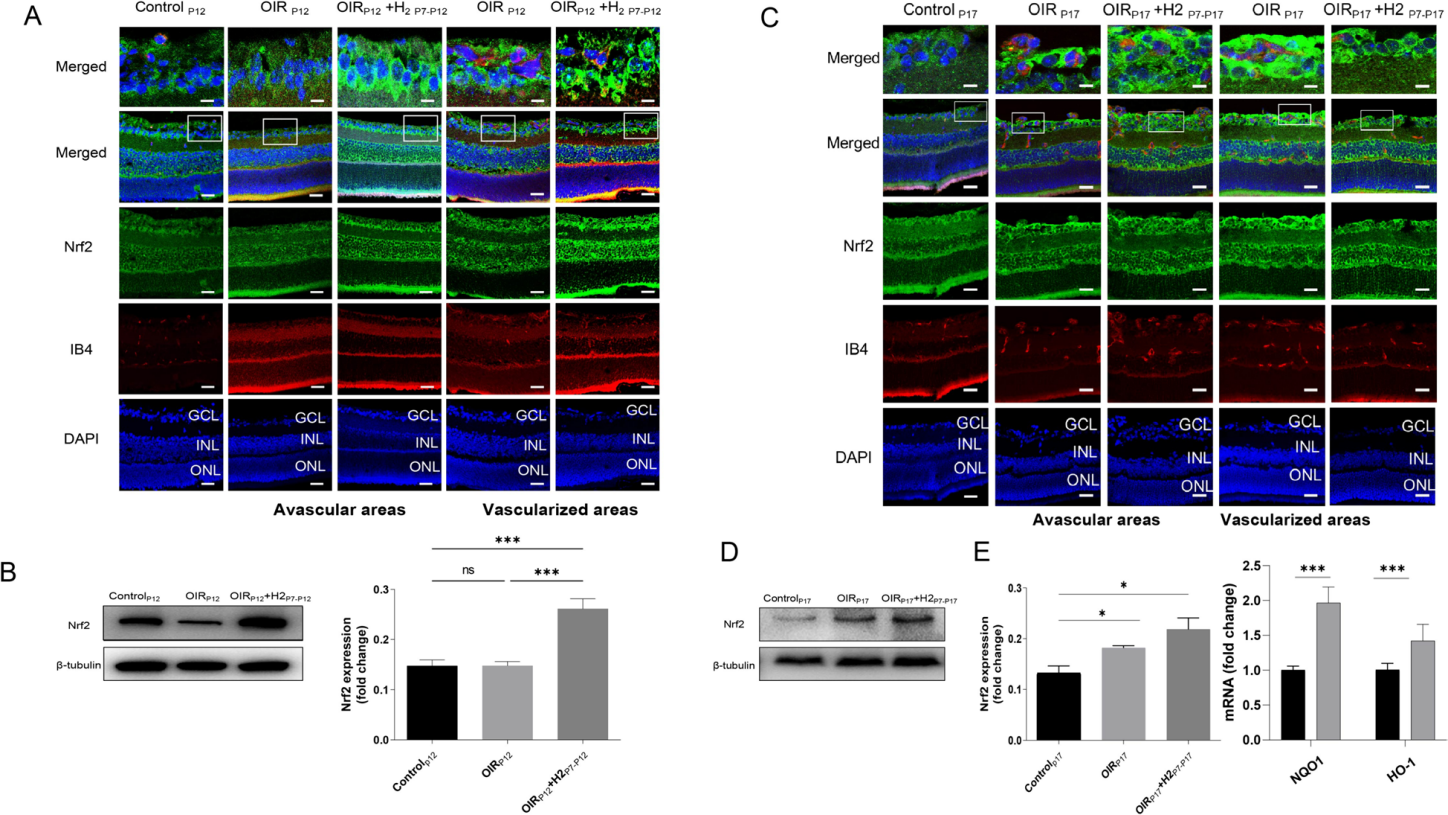
H2 promotes Nrf2 activation in OIR mice. **A-B** the level of Nrf2 expression did not differ between the OIRP12 group and control group but increased after H2 inhalation. **C-D** At P17, higher Nrf2 levels were observed in both the OIRP17 group and the OIRP17+H2P7-P17 group than in the control group. **E**. H2 treatment enhanced the expression of the main target genes of Nrf2 (HO-1 and NQO1)

Western blot results indicated that the level of *Nrf2* expression did not differ between the OIR_P12_ group and control group but increased after H_2_ inhalation (Fig. 4A, B). These results were consistent with previous studies showing that *Nrf2* was activated after the onset of ischemia. Interestingly, H_2_ application induced early *Nrf2* activation on P12, which revealed that H_2_ promoted *Nrf2* activation during the hyperoxic phase. At P17, higher *Nrf2* levels were observed in both the OIR_P17_ group and the OIR_P17_ + H2_P7–P17_ group than in the control group (Fig. 4C, D). There was a trend of higher *Nrf2* expression in the H_2_ treatment group than in the OIR group. Additionally, H_2_ treatment notably enhanced the expression of the main target genes of *Nrf2*, such as HO-1 and NQO1, in the retinas of OIR mice (Fig. 4E; 2.11-fold increase in NQO1; 1.87-fold increase in HO-1). Thus, we found that the upregulation of *Nrf2* promoted by H_2_ inhalation was sustained throughout the whole procedure. (Fig. 4A, C). These results therefore confirmed that H_2_ might exert its protective effect on retinopathy in the OIR model by promoting the activation of the *Nrf2* pathway.

### Genetic ablation *of Nrf2* impeded the protection provided by H_2_ against retinopathy in OIR mice

To determine whether *Nrf2* is required for the protective effects of H_2_ on retinal angiogenesis in OIR mice, we investigated retinal angiogenesis in *Nrf2−/−* mice treated with H_2_. According to Koichi and Wei’s study [22, 26], avascular areas in *Nrf2−/−* mice on P9 were significantly larger but similar on P12 to those in *Nrf2* +*/*+ mice. Hyperoxia treatment revealed a similar window of time where Nrf2-regulated antioxidant production was beneficial and contributed to endothelial survival.

**Fig. 5:**
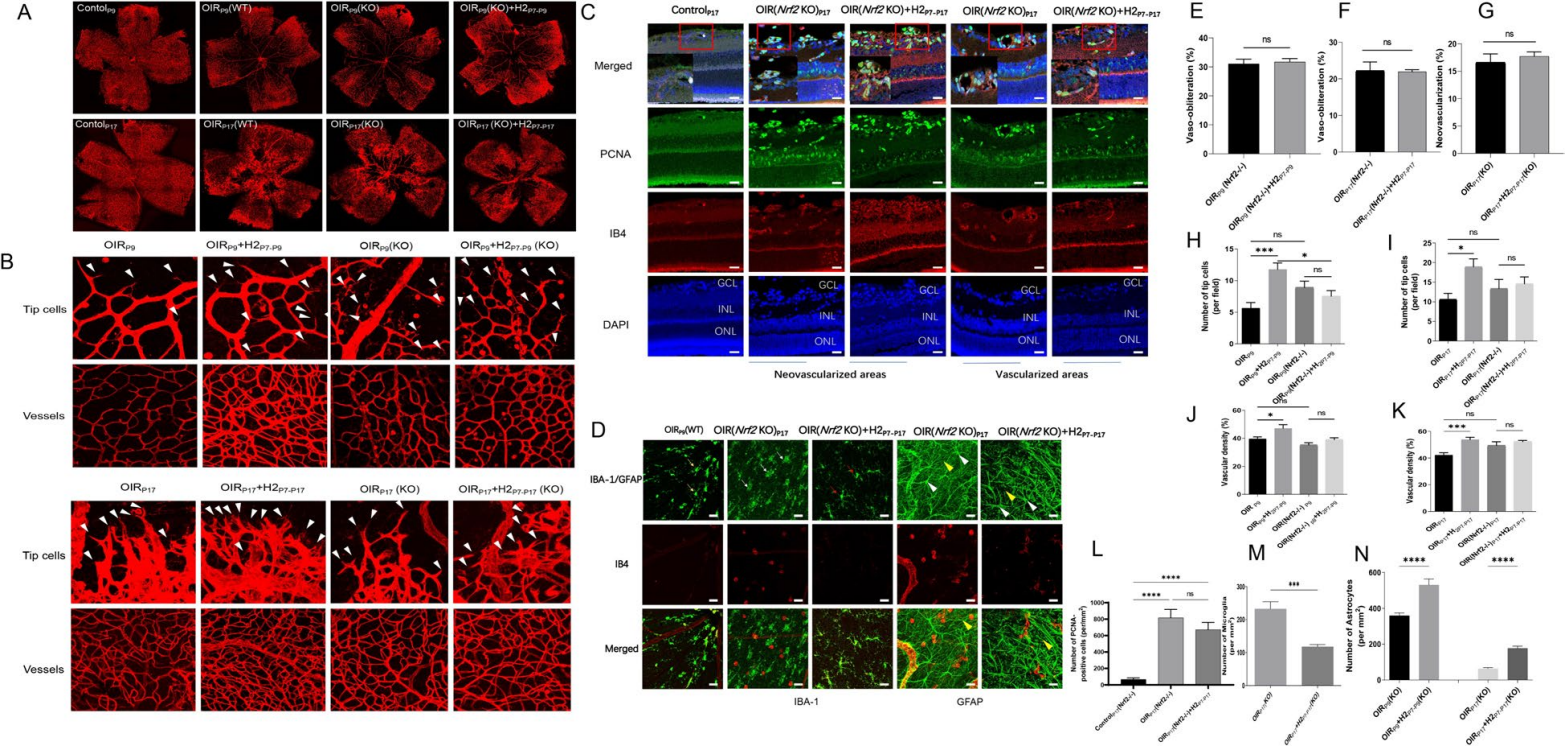
Genetic ablation of Nrf2 impeded the protection provided by H2 against retinopathy in OIR mice **A．** **E-G** H2 could not ameliorate retinal pathological neovascularization and vaso-obliteration in Nrf2−/− OIR mice. **B.H-I** The number of tip cells at the junction of the avascular and neovascular regions and the density of blood vessels in the peripheral region in each group. **D.M-N** Double labeling of isolectin B4 and GFAP showed that tip cells in the retina of the OIR+H2 group were attached to astrocytes, and their filopodia were stretched along them. They were not present in Nrf2-KO mice. **C,L.** H2 could not exert its antiangiogenic effect without Nrf2 participation. There were no differences in the expression of PCNA between the Nrf2-/-OIR and Nrf2-/- OIR+H2 groups

Thus, we chose OIR mice on P9 to estimate vaso-obliteration of hyperoxia-exposed retinas in *Nrf2−/−* and WT mice. The extent of vaso-obliteration and NVT can be determined by the ratio of the avascular/NVT area accounting for the whole-mount retina at P9 and P17. The avascular areas accounted for 32.38% ± 0.81% and 31.71% ± 1.18% of the total retinal area in the *Nrf2−/−* OIR_P9_ and *Nrf2−/−* OIR_P9_ + H_2 P7-P9_ groups, respectively, with no statistically significant difference during the hyperoxia period (Fig. 5A). During the hypoxia stage, neovascular areas and avascular areas were observed in retinas from the *Nrf2−/−* OIR _P17_ and *Nrf2−/−* OIR _P17_ + H_2_ groups. No changes in avascular or neovascular areas were found in the *Nrf2−/−* OIR_P17_ and *Nrf2−/−* OIR _P17_ + H_2_ groups. As mentioned above, *Nrf2−/−* OIR mice exhibited a remarkably higher extent of avascular retina than OIR-WT mice at P17, which is consistent with previous reports [22]. In addition, H_2_ could not ameliorate retinal pathological neovascularization and vaso-obliteration in *Nrf2−/−* OIR mice, indicating that H_2_ treatment participates in vascular remodeling through *Nrf2* activation in OIR mice. (Fig. 5A, E–G).

Next, we investigated the number of tip cells at the junction of the avascular and neovascular regions and the density of blood vessels in the peripheral region in each group. As shown in Fig. 5B, the number of tip cells and vascular density was higher by 2.08 times and 1.19 times, respectively, in the OIR_P9_ + H_2 P7–P9_ group than in the OIR_P9_ group (Fig. 5B). The number of tip cells and vascular density was higher by 1.77 times and 1.28 times in the OIR_P17_ + H_2 P7–P17_ group than in the OIR_P17_ group (Fig. 5H–K). Nevertheless, there was no significant difference in the number of tip cells or vascular density between the OIR (*Nrf2−/−)*_P9_ and OIR (*Nrf2−/−*)_P9_ + H_2P7–P9_ groups, and no differences were observed in the number of tip cells or vascular density between OIR (*Nrf2−/−)*_P17_ mice and the OIR (*Nrf2−/−)*_P17_ + H2_P7–P17_ groups (Fig. 5B, H, I). Moreover, double labeling of isolectin B4 and GFAP showed that tip cells in the retina of the OIR + H_2_ group were attached to astrocytes and that their filopodia were stretched along them,which were not present in *Nrf2-*KO mice (Fig. 5D). These results suggested that H_2_ inhalation improved revascularization and the formation of tip cells, which is guided by the astrocyte network. *Nrf2* ablation weakened the protective effect of H_2_ against retinopathy in OIR mice.

**Fig. 6:**
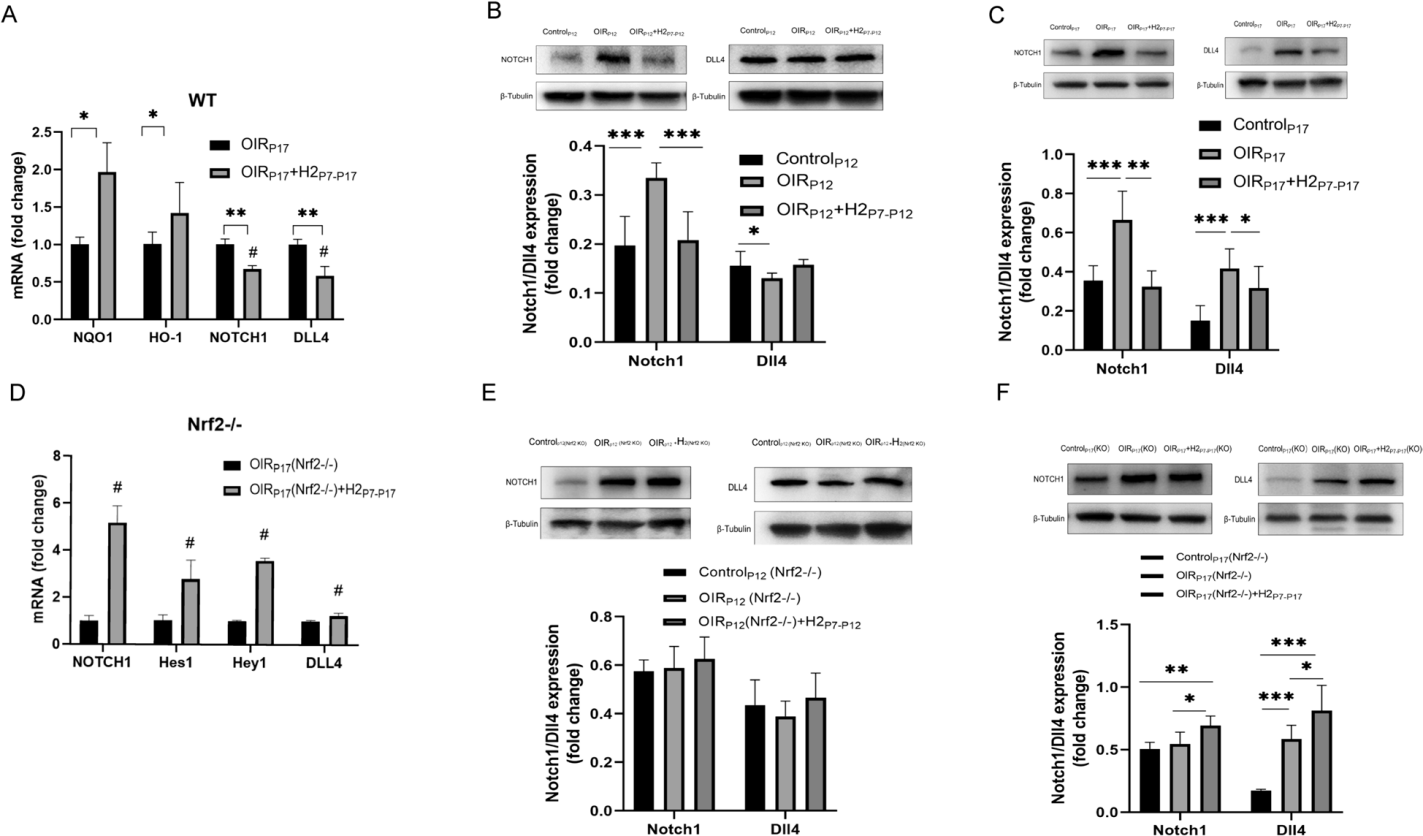
H2 promotes retinal vascular regeneration and ameliorates neovascularization of OIR mice Via Nrf2-notch axis.** A.** H2 upregulated Nrf2 target genes (NQO1 and HO-1) and downregulated Notch/Dll4 mRNA expression. Nrf2 KO abolished the effect of H2 on Notch/Dll4 expression at the transcriptional level. **B.** Dll4 and Notch expression was suppressed in the OIRP17 + H2 P7–P17 group. **C.** H2 halation suppressed the elevation in the Notch1 and Dll4 protein level of OIR mice at P17. **D.F** Nrf gene ablation abolished the suppression of Notch/Dll4 in H2-treated OIR mice. E.Notch1 and Dll4 expression have an increasing trend after H2 treatment, although with no significant difference

We further explored the expression of PCNA, which closely correlates with the proliferation activity of vessel endothelial cells. The density of PCNA/IB4-positive cells within the neovascular area in the retinas of the *Nrf2−/−* OIR and *Nrf2−/−* OIR + H_2_ groups was higher than that in the controls and even showed an increase in the normal vasculature area. The results showed that the level of PCNA in the *Nrf2−/−* OIR and *Nrf2−/−* OIR + H_2_ groups was higher by 9.54-fold and 9.44-fold, respectively, the levels in the controls (*Nrf2−/−* mice) under normoxia. In addition, as shown in Fig. 6K, there were no differences in the expression of PCNA between the *Nrf2−/−* OIR and *Nrf2−/−* OIR + H_2_ groups. These results revealed that H_2_ could not exert its antiangiogenic effect without *Nrf2* participation(Fig. 5C, L).

Moreover, due to the vital function of astrocytes and microglia, we examined whether *Nrf2* gene deletion blocked the effect of H_2_ on neuroglia. Surviving retinal astrocytes were labeled by GFAP immunostaining in *Nrf2−/−* OIR mice on P17 and exhibited finer processes and a decrease in astrocyte density in the avascular area of retinas. However, H_2_ administration preserved the distribution of astrocytes and reduced the disruption of the astrocyte template in *Nrf2−/−* OIR mice (Fig. 5D, N). In addition, microglial cells in *Nrf2−/−* OIR mice showed enlarged cell bodies and shorter processes with fewer branches. After H_2_ treatment in *Nrf2−/−* OIR mice, the proportion of amoeboid microglial cells was reduced by almost half (Fig. 5D, M). These data indicated that H_2_ could preserve the astrocyte template and inhibited the activation of microglia in the retinas of OIR mice without *Nrf2* signal pathway participation.

### H_2_ promotes retinal vascular regeneration and ameliorates neovascularization of OIR mice Via *Nrf2-notch* axis

The aforementioned results encouraged us to explore the underlying mechanism of the effects of H_2_ on the OIR mouse model. As mentioned above, increased vascular regeneration and alleviated pathological neovascularization by H_2_ were eliminated after Nrf2 knockout. It is known that the transcription factor Nrf2, well known for regulating the stress response in multiple pathologic settings, is a critical intracellular regulator in ECs for sprouting angiogenesis in vascular development through delta-like 4 (Dll4)/Notch signaling [20]. Given the critical role of Notch ligand Dll4 in negatively regulating angiogenic sprouting, we hypothesized that Dll4/Notch signaling might be modulated by Nrf2 in H_2_-induced vascular remodeling.

To confirm that inhibition of vascular Notch1 / Dll4 is a key mechanism for hydrogen-induced vascular remodeling of OIR,we observed the mRNA and protein expression levels of different groups of Notch1 and Dll4. H_2_ upregulated Nrf2 target genes (NQO1 and HO-1) and downregulated Notch/Dll4 mRNA expression. Nrf2 KO abolished the effect of H_2_ on Notch/Dll4 expression at the transcriptional level (Fig. 6A). Compared with the OIR_P17_ group, Nrf2 target genes (NQO1 and HO-1) expression was higher in the OIR_P17_ + H_2 P7–P17_ group. Accordingly, Dll4 and Notch expression was suppressed in the OIR_P17_ + H_2 P7–P17_ group (P < 0.05).

Contrary to inhibiting the Notch1/Dll4 pathway in OIR_P17_ + H_2 P7–P17_ mice,Notch and Dll4 target gene (Hes1, and Hey1) expression was higher in *Nrf2−/−* retinas with H_2_ inhalation than in those with OIR_P17(WT)_ + H_2 P7–P17_ (P < 0.05 Fig. 6D). In consistent with mRNA level expression,western blot results showed that H_2_ halation suppressed the elevation in the Notch1 and Dll4 protein level of OIR mice at P17 (Fig. 6C). In addition, compared with control_P17_ (*Nrf2−/−*) mice, Notch1 and Dll4 protein expression was upregulated in the OIR_P17_ (*Nrf2−/−*) group,even higher in OIR_P17_ (*Nrf2−/−*) + H_2_ group_,_which indicated Nrf gene ablation abolished the supression of Notch/Dll4 in H_2-_treated OIR mice (Fig. 6D, F). Next,we, examined the Notch/Dll4 protein level of OIR mice at P12. Western blot results showed that H_2_ reduce the elevation in the Notch1 protein level of OIR mice,but not Dll4. In *Nrf2−/−* OIR mice,Notch1 and Dll4 expression have a increasing trend after H_2_ treatment,although with no significant difference (Fig. 6B, E). These results suggest that H_2_ inhalation inhibited the Notch1/Dll4 pathway through Nrf2 activation. Therefore, H_2_ negatively regulates Dll4/Notch signaling to participate in retinal angiogenesis through Nrf2 activation in OIR mice.

### H_2_ suppressed notch/Dll4 and ROS via *Nrf 2* activation in vitro

**Fig. 7:**
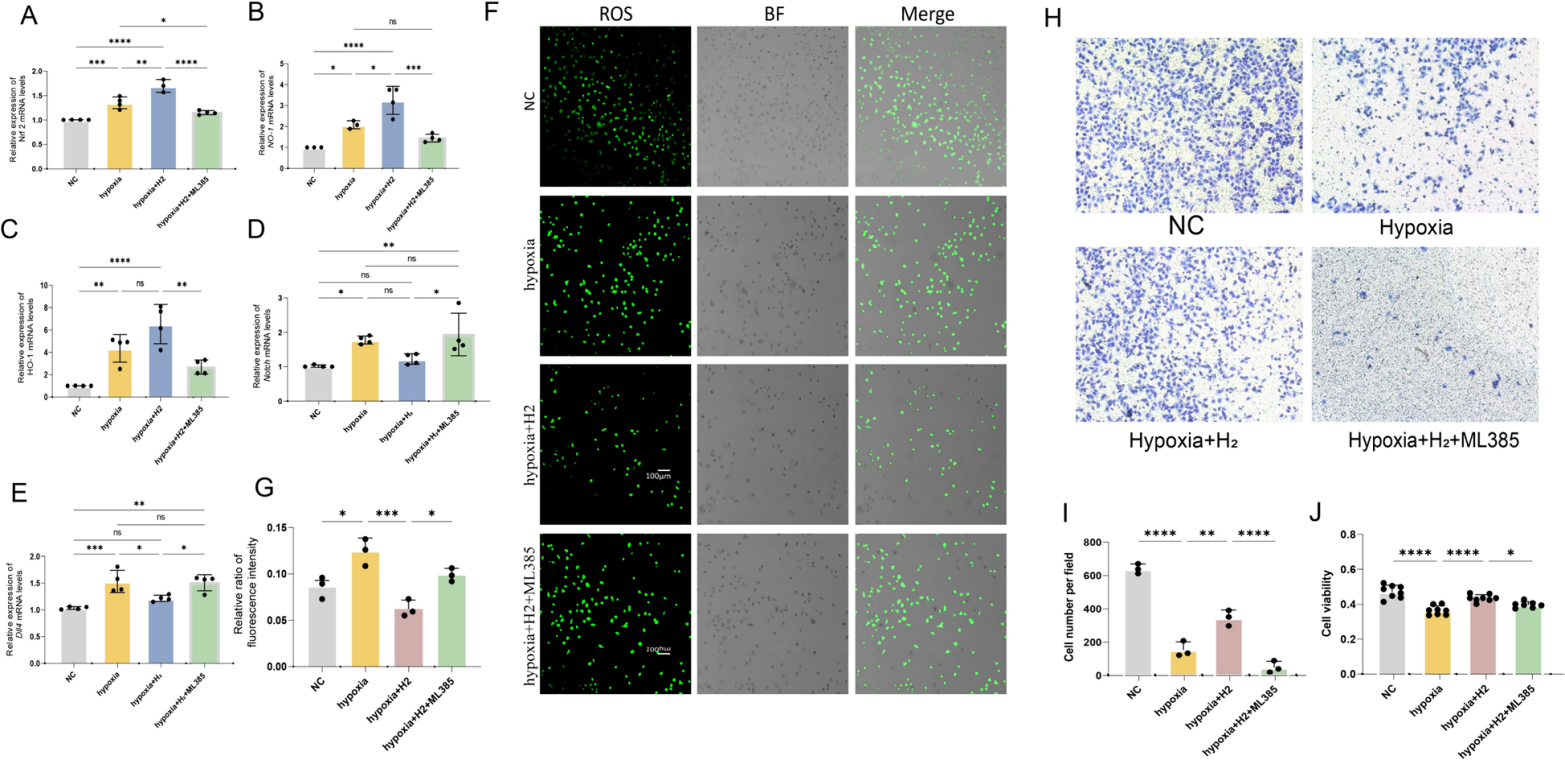
H2 suppressed notch/Dll4 and ROS via Nrf 2 activation in vitro. **A-E** an increase in Nrf2 expression and its target genes, as well as Dll4/Notch expression, in the hypoxic group compared to the normal group. Treatment with H2 further increased Nrf2 expression and decreased Dll4/Notch expression in the hypoxia + H2 group. In the presence of ML385, the combination of hypoxia and hydrogen gas resulted in decreased Nrf2 expression and increased Dll4/Notch expression. **F.G** Under hypoxic conditions, the presence of H2 decreased the intensity of reactive oxygen species (ROS) fluorescence in cells, while Nrf2 inhibition led to an increase in ROS levels

To verify whether the interaction between *Nrf 2* and Notch/Dll4 pathway and the H_2_ in regulating the Dll4/Notch axis via *Nrf 2*, we conducted an in vitro study using human umbilical vein endothelial cells (HUVECs) cultured under hypoxic conditions. We observed an increase in *Nrf2* expression and its target genes, as well as *Dll4/Notch* expression, in the hypoxic group compared to the normal group. Treatment with H_2_ further increased *Nrf2* expression and decreased *Dll4/Notch* expression in the hypoxia + H_2_ group. We determined that the maximum inhibitory concentration of the *Nrf2* inhibitor ML385 was 5 μM (Figure S2) [27]. In the presence of ML385, the combination of hypoxia and hydrogen gas resulted in decreased *Nrf2* expression and increased Dll4/Notch expression(Fig. 7A–E). These findings suggest that *Nrf2* activation negatively regulates the expression of the Dll4/Notch signaling pathway, consistent with in vivo results. Additionally, under hypoxic conditions, the presence of H_2_ decreased the intensity of reactive oxygen species (ROS) fluorescence in cells, while *Nrf2* inhibition led to an increase in ROS levels once again(Fig. 7F, G).

Based on this, it can be inferred that hydrogen protect endothelial cell function.The MTT results showed that hydrogen enhanced the inhibitory effect of hypoxia on endothelial cell growth, but this effect was reduced when *Nrf2* was inhibited. The Transwell data revealed that hydrogen significantly improved HUVEC migration, while hypoxia inhibited it. However, this effect was significantly diminished after *Nrf2* inhibition(Fig. 7G, H). This suggests that hydrogen gas promotes the proliferation of normal endothelial cells by regulating negatively the *Dll4/Notch* pathway through *Nrf2*, and it also protects these cells from damage caused by oxidative stress by activating *Nrf2* under hypoxia treatment.

### H_2_ protected retina against retinopathy of OIR mice via dual-directional regulation of HIF-1α-VEGF pathway both in hyperoxic and hypoxic phases.

In addition to Notch signaling, numerous studies showed that the *HIF-1α*-VEGF pathway was involved in retinal neovascularization [28]. Hypoxia-inducible factor 1 alpha subunit (*HIF-1α*) is a key transcription factor in cellular responses to hypoxia and plays a critical role in angiogenesis by activating the transcription of genes encoding angiogenic growth factors. Therefore, we also explored whether H_2_ could regulate the *HIF-1α*-VEGF pathway in the retina during different phases. The protein levels of *HIF-1α* and VEGF in the retinas of different groups were examined by Western blot and immunofluorescence staining.

**Fig. 8:**
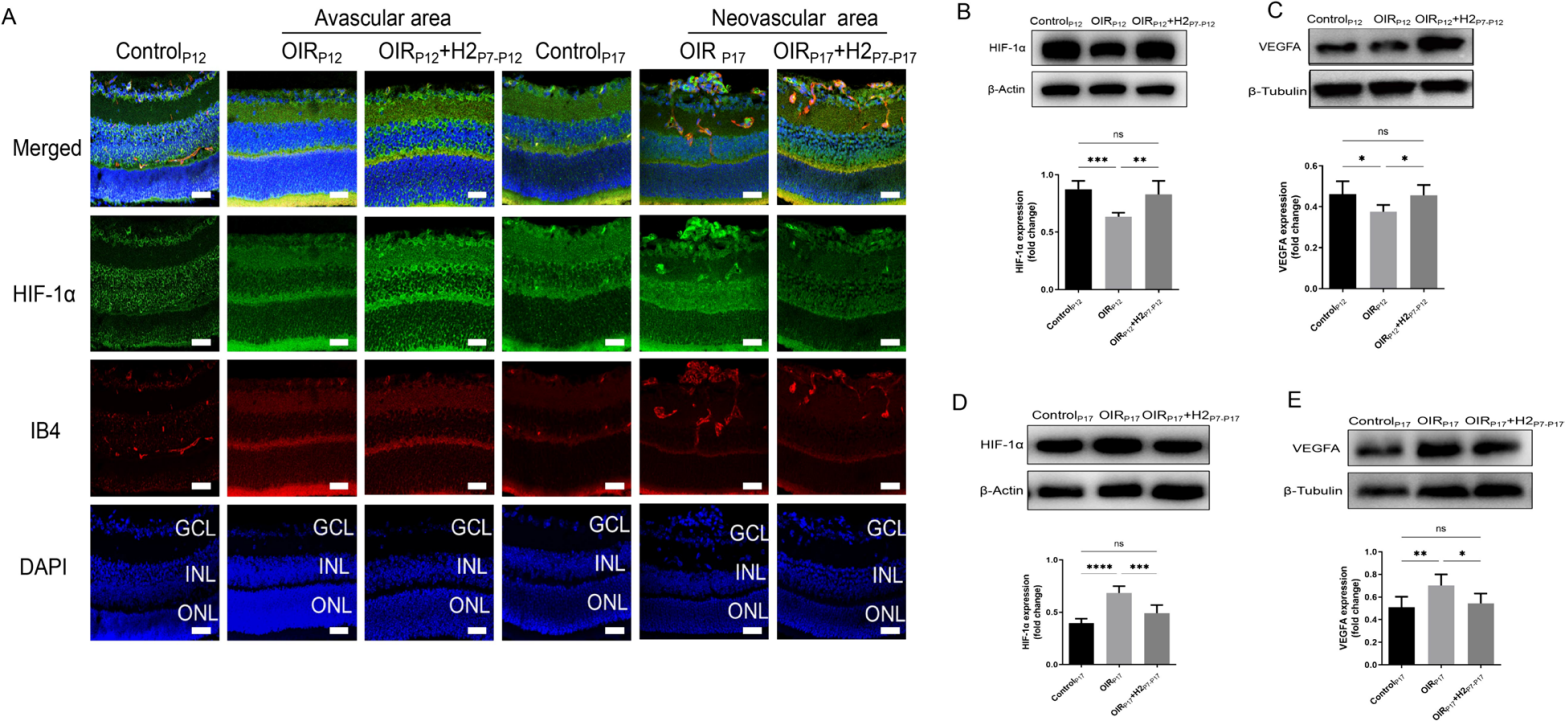
H2 protected retina against retinopathy of OIR mice via dual-directional regulation of HIF-1α-VEGF pathway both in hyperoxic and hypoxic phases. **A．**HIF-1α was expressed in both the INL and GCL in the control littermate on P12, but the expression of HIF-1α in the avascular area was suppressed in mice in the OIRP12 group. In contrast, H2 inhalation increased the expression of HIF-1α in OIR mice at P12；HIF-1α was slightly expressed in the control group at P17. Unlike the increased HIF-1α expression in the OIR group, HIF-1α expression was decreased in OIR mice on P17 after H2 treatment. **B-C** an evident decrease in HIF-1α and VEGF protein expression in the OIR group and an increase in HIF-1α and VEGF protein expression after H2 treatment. **D-E** H2 inhalation reversed the trend of increased HIF-1α and VEGF protein expression in OIR mice to the level in control littermates on P17

Our immunofluorescence staining results showed that *HIF-1α* was expressed in both the INL and GCL in the control littermate on P12, but the expression of *HIF-1α* in the avascular area was suppressed in mice in the OIR_P12_ group. In contrast, H_2_ inhalation increased the expression of *HIF-1α* in OIR mice at P12 (Fig. 8A). There was an evident decrease in HIF-1α and VEGF protein expression in the OIR group and an increase in *HIF-1α* and VEGF protein expression after H_2_ treatment(Fig. 8B, C).

*HIF-1α* was slightly expressed in the control group at P17. Unlike the increased *HIF-1α* expression in the OIR group, *HIF-1α* expression was decreased in OIR mice on P17 after H_2_ treatment (Fig. 8A). In agreement with these results, Western blot analysis revealed that H_2_ inhalation reversed the trend of increased HIF-1α and VEGF protein expression in OIR mice to the level in control littermates on P17 (Fig. 8D, E). These data suggest that in the OIR mice, hypoxia reduced the level of retinal *HIF-1α*/VEGF expression, whereas hyperoxia increased the activation of the retinal *HIF-1α*/VEGF pathway. H_2_ therapy exerts bidirectional regulation to dynamically control appropriate *HIF-1α*/VEGF expression. Therefore, we could further infer that the *HIF-1α*-VEGF pathway was also involved in the protective effects of H_2_ on the retinas of OIR mice.

## Discussion

Here, the principal findings of the present study were that molecular hydrogen exerts a substantial beneficial effect on the pathogenesis of oxygen-induced retinopathy both at the hyperoxic and hypoxic phases. Several pieces of evidence were provided to support these results. *First, *H_2_ promoted vascular regeneration and attenuated retinal vaso-obliteration by improving the development of sprouts of the normal vessels. Simultaneously, H_2_ reduced pathological neovascularization in the retina of OIR by inhibiting PCNA-positive vessel proliferation; *Second*, H_2_ exerted its neuroglialprotective effect by the preservation of astrocytic template and inactivation of microglia. The former provided the template over which endothelial cells migrate to form the retinal vascular network. The latter released excessive pro-inflammatory cytokines and consequently led to inflammatory responses, causing vascular pathology. *Third*,we uncovered key cellular mechanisms that H_2_ affected the the retinal vascular regenerationandpathologic neovascularization suppression in the OIR mice by regulating the *Nrf2 /Notch1/ Dll4* axis and *Hif-1α/VEGF* pathway in vivo and in vitro. *Last but not least,* neuroglial protection after H_2_ treatment was considered as partially independent of the *Nrf2* pathway, mainly due to H_2_’s anti-inflammatory and antioxidant effects.

Current treatments for ROP such as laser photocoagulation [10, 29], and intravitreal anti-VEGF therapy [10], target the neovascular and end-stage of the disease, and although moderately beneficial, are associated with significant sequelae including unknown systemic risks, adverse events such as retinal detachment or recurrence of ROP and compromised visual acuity. Hence, preventative and free-of-side-effect treatments that influence the fundamental pathological pathways involved in ROP are desired. As a promising medical gas, inhaled H_2_ has been widely used in clinical [30]. The numerous publications on its biological and medical benefits revealed that H_2_ reduces oxidative stress not only by direct reactions with strong oxidants but also indirectly by regulating various gene expressions. Moreover, by regulating the gene expressions, H_2_ functions as an anti-inflammatory and anti-apoptotic, and stimulates energy metabolism which was related to angiogenesis in ECs. By a clinical examination, Ono et al. showed that inhalation of 3–4% H_2_ gas reached a plateau at approximately 10–20 μM in the arterial and venous blood, respectively, in about 20 min and affected no physiological parameters, suggesting no adverse effects [15]. However, to our knowledge, no data are available regarding the effects of inhaled H_2_ on retinal vascularization in healthy animals. Hence, the first purpose of this work is to screen for adverse effects of inhaled H_2_ on normal retinal developmental angiogenesis in healthy mice. Several lines hint that H_2_ indeed shas no adverse effect on retinal angiogenesis during postnatal development. The retinal vessels of mice with H_2_ inhalation display normal retinal vascularization, vascular vessel density, and sprouting activity (number of tip cells).Moreover, healthy astrocytes form a reticular network that provides a substrate for migrating endothelial cells in H_2_ inhalation mice, where an astrocyte template is required for normal retinal angiogenesis and vessel patterning. In addition, because of safety and clinical application considerations, we choose the concentration of H_2_ gas 3 ~ 4%, which was the safety concentration under the explosive concentration of H_2_ gas in the mixture of H_2_ gas and air as 4–75% [31].

Next, whether H_2_ exerts a protective effect on ocular vascularization remains controversial. Some studies showed that the effects of H_2_ are remarkable in reducing retinal tissue damage in a retinal ischemia–reperfusion injury [32]. On the contrary, Zhang et al. found that hydrogen-rich saline therapy (at the dose of 10 ml/day, applied between P12 and P17) did not inhibit retinal neovascularization in OIR [33]. In favor of Pros, our present study suggested that H_2_ could prevent pathologic neovascularization and promote angiogenesis in the ischemia retina of OIR models.Several lines of evidence indicate that H_2_ treatment significantly reduced the size of both retinal neovascular and avascular areas, and the number of pre-retinal neovascular cell nuclei. It also inhibited the proliferation of endothelial cells within the neovascular area. Furthermore, H_2_ reversed the pathological change of astrocytes and preserved an astrocytic template for the retinal vasculature.

Number of previous studies have paid attention to treating ROP in phase II OIR when retinal dysplasia occurs [21]. However, Sapieha et al. pointed that the extent of vaso-obliteration in phase 1 OIR predicted the degree of neovascularization in phase II OIR [34] indicating that early treatment may be of most benefit.In agreement with their views, we believe that preterm infants with multiple risk factors for ROP should have early targeted treatment, which will effectively prevent the risk of increasing largely proportions of severe neovascularization in the future.In line with those results, our study showed that inhaled H_2_ reduced vaso-obliteration and neovascularization to the maximum extend during the whole intervention. This was the situation with H_2_ inhalation, which although reducing neovascularization when administered at the commencement of the neovascular phase II of OIR, was more efficacious when treatment began at phase 1 OIR. Thus, we suggest H_2_ as an alternative or additional strategy to overcome the side effects of VEGF-A blockade for the treatment of ischemic retinopathies such as ROP, PDR, and retinal vascular occlusive diseases, even intervention at an early stage.

Our fundamental question was how H_2_ can promote directional angiogenesis and the building of organized blood vessels that grow into the avascular region while suppress the occurrence of neovascularization out of the retinal surface under hyperoxia and ischemic conditions.Previous studies evaluating the effect of Nrf2 on the retina and retinal development have largely been concerned with Nrf2’s direct regulation of angiogenesis, which appears to be strongly context-dependent [20, 26].In this study, we explored that Nrf2 signaling highly activated by H_2_ was essential for revascularization enhancement and neovascularization suppression in OIR mice. The effect of early H_2_ treatment on vaso-obliteration was particularly striking and confirmed when vaso-obliteration is maximal in phase I OIR mice. Validation of inhaled H_2_ ability to reduce vaso-obliteration and hence promote normal angiogenesis in phase I OIR was shown with an increase in the protein levels of Nrf2. More worsen vaso-obliteration was observed even without improved after H_2_ inhalation in *Nrf2−/−* mice. Our results find that Nrf2 strongly activation by H_2_ forward from the onset of ischemia (P12) to the hyperoxic phase (P7–P12). These results are in agreement with studies utilizing Nrf2 knockout mice, which exhibited exacerbated vaso-obliteration in OIR [20]. Moreover, of relevance is evidence that Nrf2 is a critical intracellular regulator in endothelial cells for sprouting angiogenesis in vascular development through delta-like 4 (Dll4)/Notch signaling inhibition [20].

According to Koichi Uno and Wei’s study [20, 22, 26], avascular areas at P12 were significantly smaller than those at P9, Also no significant difference in avascular areas was found between *Nrf2−/− *and WT mice at P12.Consistent with this view, *Nrf2* was activated after the hyperoxic phase.Thus, we chose OIR mice on P9 to estimate vaso-obliteration of hyperoxia-exposed retinas in *Nrf2−/−* and *Nrf2* + */* + mice. Our results indicated that more severely avascular areas were showed in *Nrf2−/−* mice at P9, which suggested this protection by *Nrf2* occurs in a specific window of time was beneficial and contributed to the endothelial survival under hyperoxia condition.H_2_ prolonged the protection timing window through Nrf2 activation from P9 to P12.

In phase II OIR, exposure to room air provides the ischaemic stimulus in the retina to promote neovascularization. Previous studies have found that *Nrf2* in ECs is particularly critical in the suppression of pathologic retinal neovascularization and preservation of vascular function. Deletion of *Nrf2* led to an exacerbation of angiogenesis in peripheral ischemia in limbs and lungs [35, 36]. Activation of neuronal and vascular *Nrf2* can largely enhance vessel regrowth into the avascular zone [22].Consistent with this finding, our results showed that H_2_ suppressed pathologic neovascularization as well as promoted revascularization in the developing retina of OIR models was accompanied by an increased in *Nrf2* and its target genes *NQO1, HO-1* in our study. Activation of the *Nrf2/HO-1* axis attenuated ischemia–reperfusion injury and improved neurological function [37]; Accordingly, we identified restrained *Dll4/Notch* signaling by activation of *Nrf2*. Global *Nrf2* deficiency blocked the protection effect of H_2_, suppressing retinal revascularization and increasing pathologic neovascularization with *Dll/Notch* up-regulation in OIR mice.The findings from in vivo experiments strongly confirm the notion that activating Nrf2 leads to the beneficial effects of reducing reactive oxygen species (ROS) and inhibiting the Dll4/Notch signaling pathway.

On the other side, we pay attention to *Nrf2* influencing retinal vasculopathy by focusing on glial cells that play an indispensable role in supporting the retinal vasculature [38–40] The glial cell types of the retina consist of macroglia, including Müller cells as well as astrocytes, and microglia [41]. First, retinal astrocytes, located within the nerve fiber layer, are critical for the proper development of the retinal vasculature because of their supporting functions. In retinal development, astrocytes by their physical contact with the vasculature and mediation of the extracellular assembly of matrices provide a template for physiological angiogenesis^[55]^. In the early stages of O_2_-induced retinopathy, hyperoxia results in injury to number of astrocytes and vaso-obliteration [42]. It is suggested toapply strategies to restore astrocytes which are suggested as an approach to treat OIR [42]. Our finding revealed that in phase I OIR, H_2_ prevented the dissociation of astrocyte end feet from retinal blood vessels.*Nrf2* deletion aggravated the reduction of astrocytes and blocked astrocytes cytoprotection of H_2._In phase II OIR, our results showed that not only the number of astrocytes declined but also morphology and distribution altered in the retina after oxygen-induced injury.Inhaled H_2_ was proved to restore the integrity of the astrocyte network; therefore, the formation of tip cells was improved, and the size of vascular tufts was reduced. Tip cells are known as a specialized subset of endothelial cells at the tips of vascular sprouts, and play a motile and navigate role in angiogenesis. In the case of pathological conditions, they locate at prospective reperfusion sites in the central, remodeling zone [43, 44]. Our result showed an increase in the number of tip cells within the avascular area colocated with astrocytes in the retina of OIR after H_2_ inhalation revealing that H_2_ could improve the revascularization of the avascular area of the retina of mice in the OIR group. In *Nrf2−/−* mice, the number of astrocytes was decreased, which supported that Nrf2 took part in astrocytes protection. Interestingly, H_2_ inhalation could reverse the damaged astrocytic template in *Nrf2−/−* mice at P17.

Our results agree with the idea that amelioration of oxidative stress imbalance in astrocytes may assist in reducing vaso-obliteration and neovascularization partially rely on the H_2_ effect of antioxidant which was independent of the Nrf2 pathway; Another,up-regulation of the Nrf2-responsive antioxidant genes (*NQO1, HO-1*) were found in the retina of OIR at P12 and P17, reversed by H_2_ inhalation. These results were supported by our studies in vitro.And, *Nrf2* deletion aggravated the reduction of astrocytes and blocked astrocytes cytoprotection of H_2_.These findings were confirmed by the previous report of cultured astrocytes that *Nrf2* activation aslo ameliorate the oxidative stress imbalance via *Nrf2* activation is central to the viability of astrocytes.

Sencond, microglia cells are resident immunocompetent cells in the retina that is the extension of the CNS [45]. Retinal microglia are activated by producing plentiful of inflammatory cytokines during the vaso-obliteration phase. The latter sustains microglial activation and exerts microvascular injury [46]. For the ischemia phase, large numbers of amoeboid microglia aggregate in and around neovascular tufts when the vascular repair is under way at P17 [47].Of note, microglial homeostasis restoration may be potentially useful in future treatment options for ischemic retinopathies. This is in good agreement with our finding that the density of retinal microglia increased and they turned into an activated state in response to oxygen-induced injury. Moreover, we have observed that microglial cells in the peripheral neovascular area were attached to the vascular tufts with the disordered distribution. Thus, it could be concluded that microglia are closely involved in the pathological processes of oxygen-induced retinopathy. Of note, H_2_ mitigated neuroinflammation probably through modulating glial activation. Previous studies have provided evidence that angiogenesis, under both physiological and pathological situations, and inflammatory responses, are two processes coordinate with each other, and they share multiple signaling mediators in common [48]. Accumulation of immune cells activates angiogenic responses in coordination with the inflammatory stimuli, usually facilitating the release of proangiogenic molecules to formulate neovascularization inducing vascular leakage. As we have mentioned above, microglia, one of the most important immune cells in the retina, were activated and increased in population in the retina following oxygen-induced injury.

Our study indicates that, unlike anti-VEGF-A therapies,H_2_ has been proven to be anti-inflammatory, anti- neovascularization and pro-revascularization.H_2_ inhalation can induce healthy and functional angiogenesis and consequently prevent retinal neuroglia injury due to ischemic injury, as evidenced by vascular sprouting, and astrocytes, microglia restored in OIR mice treated with H_2._ Accumulating evidence indicated that suppression of VEGF-A signaling, which is currently being used for the treatment of patients with neovascular retinal diseases, has the local reactions and systematic side effect. Thus, we suggest H_2_ as an alternative or additional strategy to overcome the potential side effects for the treatment of retinal vaso-obliteration and ischemic retinopathies such as ROP, PDR, and retinal vascular occlusive diseases.

There are, however, some limitations to our study. We could not monitor hydrogen levels in the plasma and retina in real-time. Additionally, other significant signaling pathways may contribute to H_2_’s protective effects against retinal neovascularization. Although the oxygen-induced retinopathy (OIR) model is frequently used to study experimental neovascularization, it may not fully represent human neovascular eye conditions. For instance, the resolution of neovascularization observed in later stages of OIR seldom occurs in human retinopathy of prematurity (ROP).

In summary, our results indicate that inhaled H_2_ can rescue retinal ischemia by inducing normal angiogenesis and suppressing pathologic retinal neovascularization in the entire retina. Of note,H_2_ also release vaso-obliteration in phase 1 OIR through angiogenesis promotion. Simultaneously,it preserves the neuroglia function and homeostasis. This implies that H_2_ can be established as a treatment strategy that focuses on the true recovery of the injured organ, rather than merely delaying disease progression.

### Supplementary Information

Supplementary Material 1.

Supplementary Material 2.

Supplementary Material 3.

## Data Availability

The original contributions presented in the study are included in the article/ supplementary material. Further inquiries can be directed to the corresponding authors.
