# Supplementary figures and images for: Molecular hydrogen promotes retinal vascular regeneration and attenuates neovascularization and neuroglial dysfunction in oxygen-induced retinopathy mice
Source: Biol Res. 2024 Jun 24;57:43. doi: 10.1186/s40659-024-00515-z (PMC11194953; doi:10.1186/s40659-024-00515-z)

## Slide 1
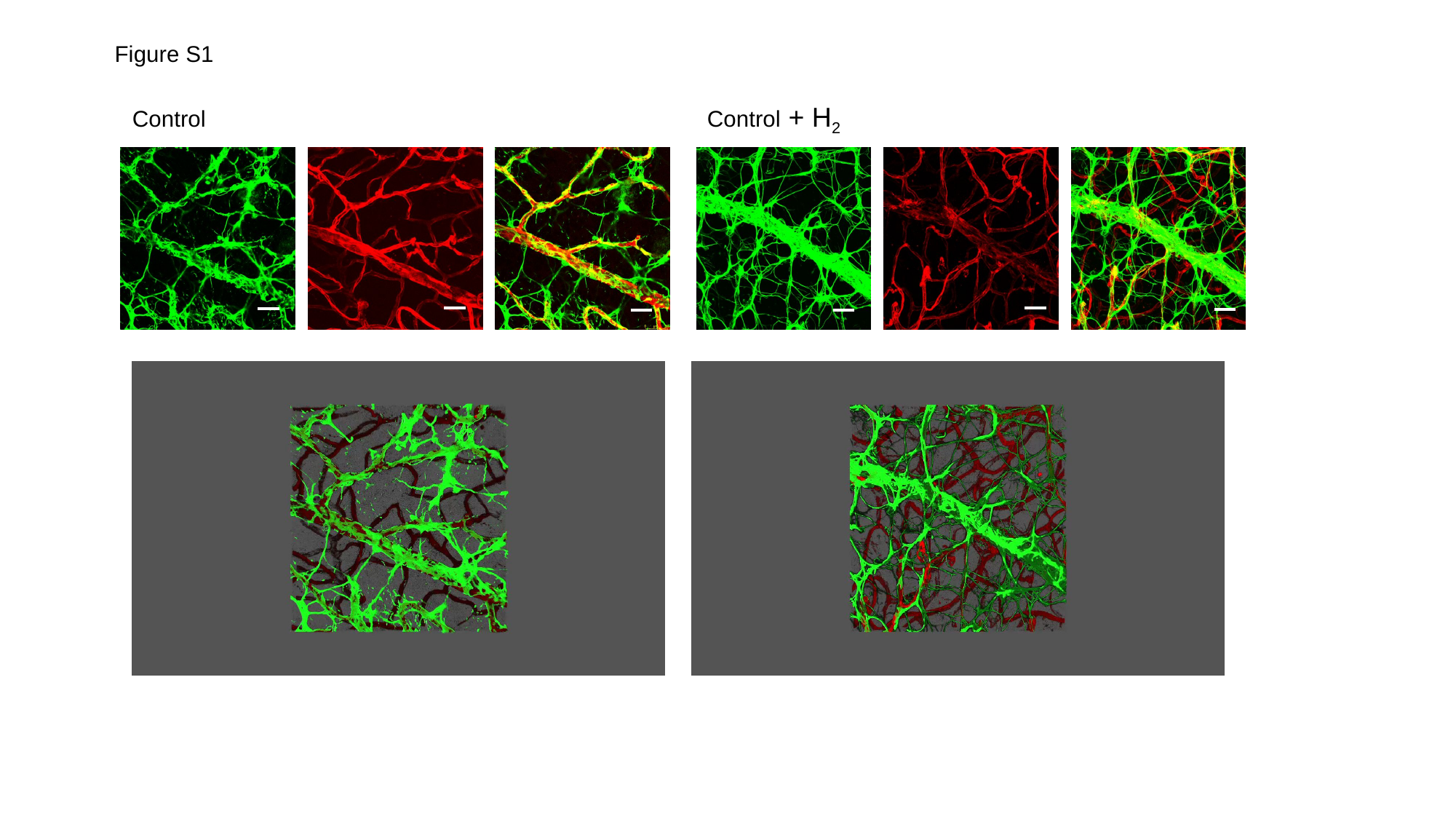

Figure S1
Control
Control + H2

Supplement: Supplementary file 1 — Supplementary Material 1. [file 40659_2024_515_MOESM1_ESM.pptx]

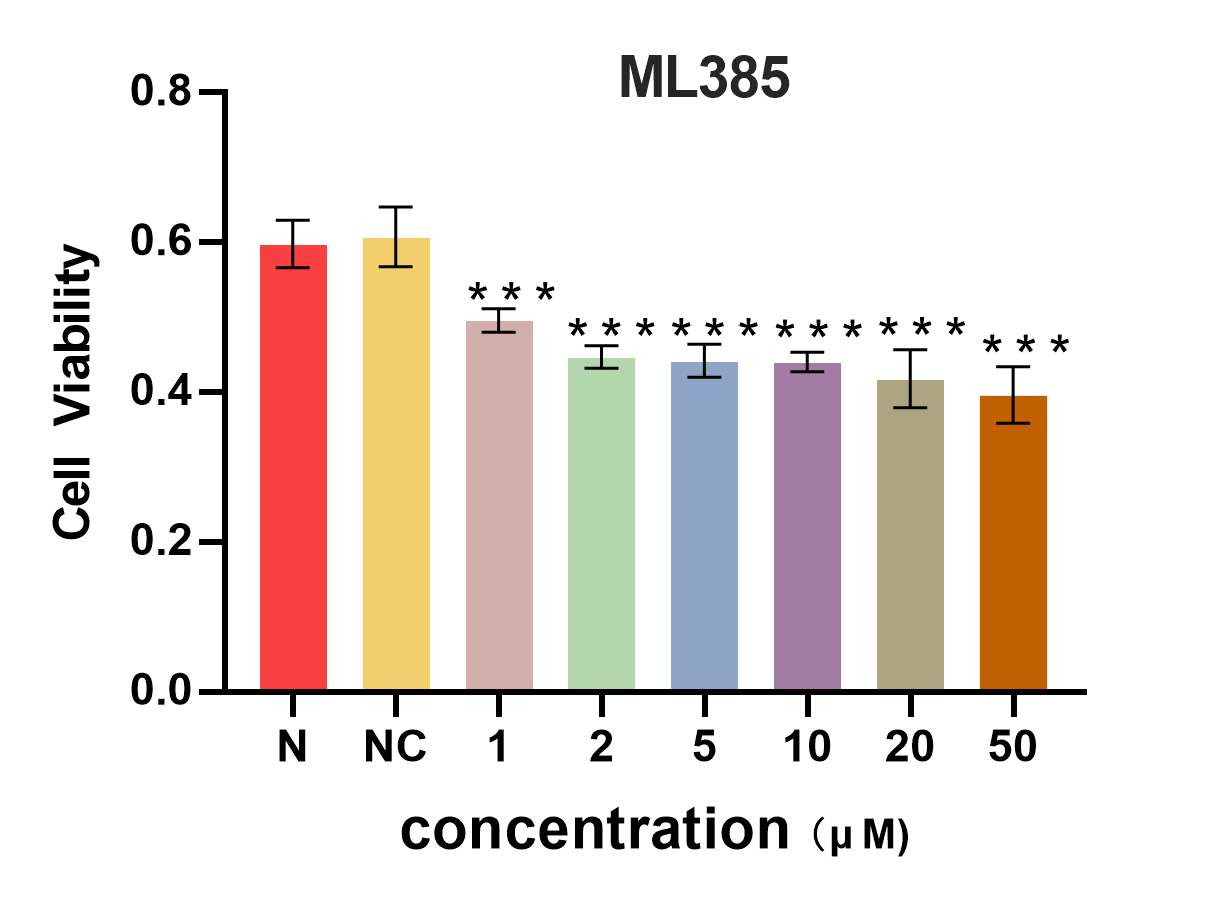

Supplement: Supplementary file 2 — Supplementary Material 2. [file 40659_2024_515_MOESM2_ESM.tif]
